# An illustrated catalogue of Rudolf Sturany’s type specimens in the Naturhistorisches Museum Wien, Austria (NHMW): deep-sea Eastern Mediterranean molluscs

**DOI:** 10.3897/zse.94.20116

**Published:** 2018-01-03

**Authors:** Paolo G. Albano, Sara-Maria Schnedl, Anita Eschner

**Affiliations:** 1 Department of Palaeontology, University of Vienna, Althanstrasse 14, 1090 Vienna, Austria; 2 Natural History Museum, Third Zoological Department, Burgring 7, 1010 Vienna, Austria

**Keywords:** Type specimens, Pola expeditions, deep-sea, Gastropoda, Bivalvia, Eastern Mediterranean Sea, Adriatic Sea, Greece, Croatia, Rudolf Sturany, Monterosato

## Abstract

The “Pola” expeditions were the first to explore the deep Eastern Mediterranean Sea in the 1890s. They remained the most intense surveys in that area for a century and constitute today a fundamental baseline to assess change in the basin, whose fauna is still inadequately described. Solid taxonomic foundations for the study of deep-sea organisms are needed and we here contribute by revising the name-bearing types of mollusc species introduced by Rudolf Sturany on the basis of the “Pola” material from the Eastern Mediterranean Sea stored in the Natural History Museum in Vienna. Sturany introduced 15 names (*Marginella occulta* var. *minor* Sturany, 1896 shall not be considered as the introduction of a new name). He described and established two manuscript names by Monterosato: *Jujubinus igneus* and *Pseudomurex ruderatus*. The genus *Isorropodon* was also introduced together with its type species *I. perplexum*. For each name, we list the available type material, provide the original description and a translation into English and illustrate the specimens in colour and with SEM imaging.

## Introduction

The second half of the 19^th^ century was an exciting period for marine exploration: most of the seafaring nations of the time sent out expeditions to investigate the sea. The Austro-Hungarian monarchy did likewise, planning a geographically restricted but intense series of expeditions to the Eastern Mediterranean and the Red Sea (Schefbeck 1996). These expeditions were the joint effort of the important research institutions of the time: The Imperial Academy of Sciences (now the Austrian Academy of Sciences), the Imperial and Royal Court Museum of Natural History (now the Natural History Museum Vienna (NHMW)) and the institutes of Zoology and Chemistry of the University of Vienna. These institutions provided the equipment, knowledge and personnel to plan and successfully conduct the expeditions. The Imperial and Royal Navy provided the ship, the crew and the commander-in-chief Admiral M.B. Sterneck, who had a soft spot for marine biology being a collector of shells and algae since an early age. He proved to be a major player in stimulating and enabling the four expeditions that were organised to the Eastern Mediterranean between 1890 and 1893 and the fifth expedition which surveyed the southern Adriatic Sea in 1894.

The Mediterranean deep sea is remarkably unexplored in comparison to its coastal areas even if it hosts several habitats that can represent biodiversity hot spots, a large share of endemic species and an estimated 66% of species still to be discovered (Danovaro et al. 2010). The “Pola” expeditions were not only the first, but also among the most intense surveys of the Eastern Mediterranean until the 1990s, when the Israel Oceanographic and Limnological Research (IOLR) started the deep-water exploration off the Israeli coast (Galil 2004). The “Thor” (1910), “Meteor 5” (1987), “Meteor 25” (1993) and “Poseidon” (cruise 201/2, 1994) contributed in-between with smaller-scale efforts.

The deep-sea Mediterranean ecosystem is also under siege from several anthropogenic stressors. As an example, climate change may affect the thermohaline circulation reducing its oxygenation (Danovaro et al. 2001) and altering the energy transport from surface waters to the seafloor (Smith et al. 2009). Furthermore, commercial fishing is extending into ever deeper waters with little appreciation of its effects on such a delicate ecosystem (Danovaro et al. 2010). In this context, the “Pola” expeditions constitute a fundamental baseline to assess change in the Eastern Mediterranean. Although not always recognized as such, the NHMW and the other institutions that preserve similar historical samples are a strategic asset in global change research (Johnson et al. 2011, Lister et al. 2011, Albano et al. 2014, Dayan and Galil 2017).

Our aim is to contribute to a solid taxonomic foundation for the study of deep-sea organisms. In particular, we revise the name-bearing types of mollusc species introduced by Rudolf Sturany (malacologist at the NHMW between 1889 and 1922 (Stagl 2012)) on the basis of the “Pola” samples from the Eastern Mediterranean Sea, following the recommendation of the International Commission on Zoological Nomenclature to publish lists of types housed in institutions (ICZN 1999, 72F.4). Similar to the previous effort on Red Sea gastropods (Albano et al. 2017), we also provide detailed illustrations of the type specimens and their diagnostic characters.

## Materials and methods

The “Pola” material is entirely stored in the NHMW (Stagl et al. 1996). Type series of Sturany’s species were segregated. Most species were represented by holotypes or very small series. In the latter case, we identified the syntypes best matching the original description but refrained from any lectotype designation following recommendation 74G of article 74.7 of the International Code of Zoological Nomenclature (“ICZN Code” in the following text) (ICZN 1999). Some names were termed as varieties of other species. Whenever Sturany clearly clarified the intention of introducing a new name, such names are considered to be of subspecific rank following the article 45.6.4 of the ICZN Code. A taxon list in alphabetical order with page and figure numbers in this paper is provided in Table 1. Any citation to the International Code of Zoological Nomenclature (ICZN 1999) should be considered to its online version which includes all recent amendments.

**Figure 1:**
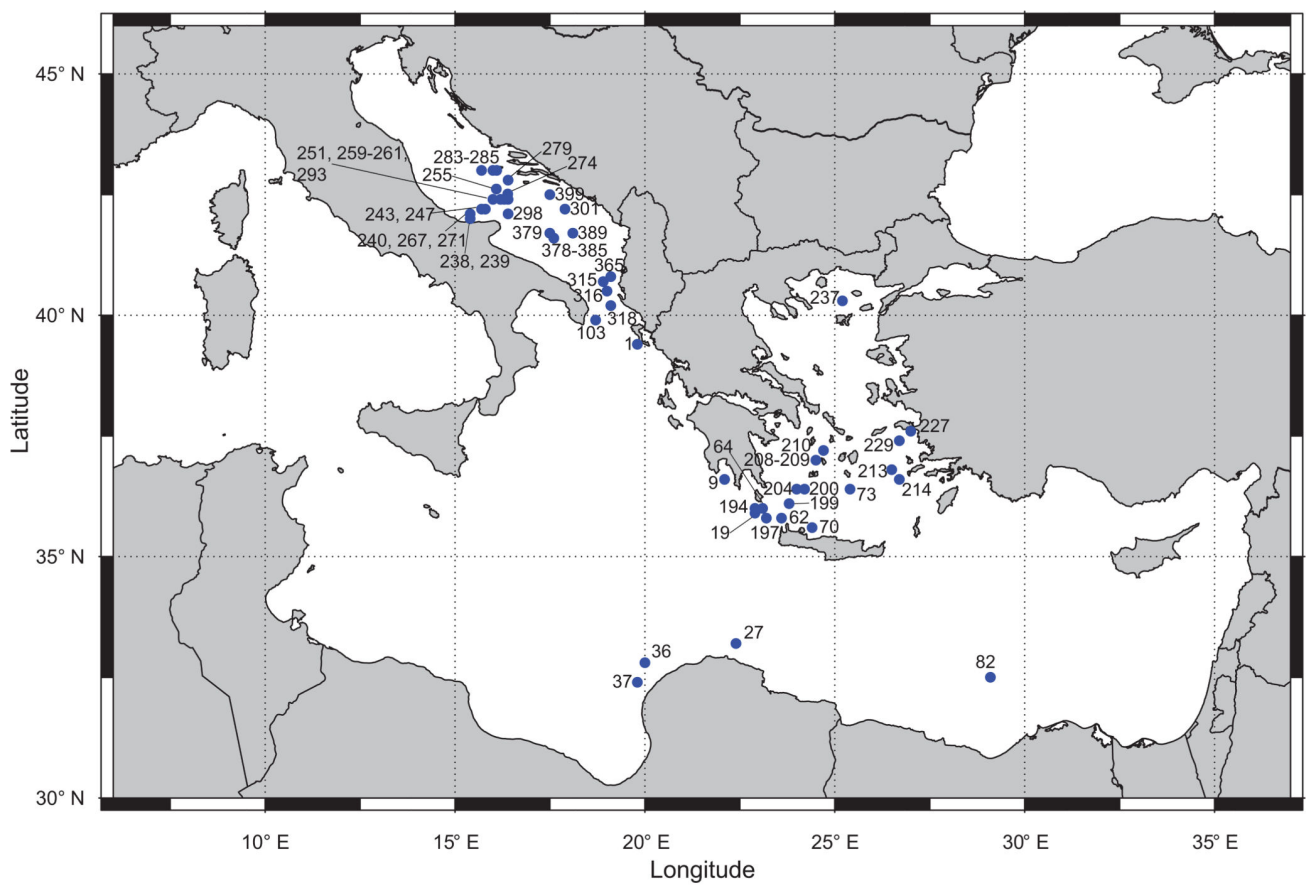
Map of the stations sampled by the “Pola” expeditions to the Eastern Mediterranean (1890-1893) and the Southern Adriatic Sea (1894) which provided molluscan samples and reported in Sturany (1896).

For each species, we provide references to the original description and figure, the original localities, a list of the type material, the original description and its translation into English. All inventory numbers provided refer to the Mollusca collection of NHMW. The systematic arrangement follows Bouchet and Rocroi (2005) and Bouchet et al. (2010) for gastropods and bivalves, respectively. The reassessment of the current taxonomic status of Sturany’s names is beyond the scope of this paper and we relied on the published literature to add comments in this regard. The expeditions surveyed several localities in Greece and Croatia (Fig. 1) whose names were stated in Italian; in our translation, we also use the modern Greek and Croatian names (Table 2).

The NHMW hosts a large collection by Monterosato that was a fundamental reference for Sturany to identify his material. This collection was purchased in 1889, just the year when Sturany started to volunteer in the mollusc collection. The acquisition from December 1889 reads: “*Shells of the Mediterranean Sea ca. 2082 sp.– collection of the zoologist Monterosato, purchased for 500 fl. Ö.W*.” [translated]. In 1897, Sturany – now assistant of the collection – wrote the complete inventory: 2113 inventory numbers altogether. Unfortunately, hardly any of the original labels are preserved within this collection nor is the original list, but Sturany had apparently transcribed all names very carefully.

Some names had been written on labels but never formally described by Monterosato. Such manuscript names have either been described by Sturany (e.g., *Jujubinus igneus*) or rendered available by redescription and illustration (e.g., *Pseudomurex ruderatus*).

Photos were mostly shot with a Nikon SMZ25 microscope; large shells were photographed with a Canon 350D camera, a 50 mm lens and extension tubes. SEM images were taken with a JEOL JSM-6610LV, using low vacuum without any coating. Specimen measurements have been added for holotypes. The map in Fig. 1 was drawn with the oceanmap package (Bauer 2017) in the statistical programming environment R, version 3.3.3 (R Core Team 2017).

## Systematic list of taxa

**Family Trochidae Rafinesque, 1815** 

***Jujubinus igneus* Sturany, 1896 ex Monterosato ms.**

Fig. 2

Sturany 1896: 28, plate II, figure 45.

**Original locality.** Station 260, at Pelagruža, Croatia, 42°23'3"N, 16°21'50"E, 128 m.

**Type material.** NHMW 72399: 3 syntypes (specimens preserved in ethanol, but apparently without the animal inside).

**Additional material.** NHMW 27441: 8 shells, Palermo, Italy (Monterosato coll.).

**Original description.** Von Station *260 (128 m).

*Die vorliegenden drei Exemplare sind circa 7,5 mm hoch und 5,7 – 6 mm breit. Von den 8 – 9 Umgängen sind die oberen rosenroth, die übrigen zart olivengrün mit rosenrothen und hellgelben Flecken oder Strichen. Die Spiralstreifen bestehen aus Reihen von groben Körnchen und zwischen ihnen stehen derbe Querstreifen. Die Zahl der Spiralstreifen ist von der vorletzten Windung an bis hinauf zum Embryonalgewinde 5, ein Hervortreten des über der Naht stehenden Streifens als Wulst, wie dies hauptsächlich für die Art* Tr. erasperatus *Penn. charakteristisch ist, ist kaum wahrnehmbar. Um den verdeckten Nabel herum verlaufen concentrisch 7 gelb und rosenroth gefleckte Rippen und ausserdem noch nahe dem Kiele der letzten Windung 1–2 schwächere ungefleckte Kreise. Die Querstreifung hier auf der Unterfläche des Gehäuses ist zarter und enger als die auf der Oberseite sichtbare*.

*Diese Beschreibung passt im Allgemeinen auch auf die von March. Monterosato mit* Jujubinus igneus *bezeichneten Exemplare, die sich in der Sammlung des Hofmuseums befinden, und deshalb habe ich diesen Collectionsnamen auch hier für die Aufschrift gewählt. Es wären für die Monterosato-Exemplare nur noch die lebhaftere rothe Färbung (Zurücktreten der olivgrünen Farbe und Prävaliren von Gelb und Rosenroth) zu erwähnen und das deutlichere Hervortreten des Suturalwulstes, der hier lebhaft roth und gelb gefleckt ist*.

*Ich möchte* Tr. igneus *Monteros. in coll. als eine Varietät von* Tr. exasperatus *Penn. hinstellen, wobei ich der von Bucquoy, Dautzenberg und Dollfus in dem Werke „Les Mollusques marins du Roussillon,u p. 362–369 ausgesprochenen Auffassung der beiden einander verwandten Arten* exasperatus *Penn. und* striatus *L. folge, muss jedoch auch bemerken, dass diese Art (nämlich* exasperatus*) mit Rücksicht auf die dort namhaft gemachte Synonymie mit* Trochus (Zizyphinus) exiguus *Pulteney (Kobelt, Prodr.p.238; Carns, Prodr. p.259) zusammenfällt*.

*Brusina und Stossich führen* Tr. exiguus *Pult. a1s* crenulatus *Brocchi für die Adria an*.

**Translation.** From station *260 (128 m).

The three specimens at hand are approximately 7.5 mm high and 5.7 – 6 mm wide. The uppermost of the 8 – 9 whorls are pink, the others are pastel olive green with pink and bright yellow marks or lines. The spiral cords consist of tubercled rows and rough horizontal stripes in-between. There are five spiral cords counting from the penultimate whorl up to the protoconch. There is hardly any prominent varix above the suture which is a typical feature in *Tr. exasperatus* Penn. Seven concentrically blended yellow and rose spotted cords frame the covered umbilicus. Moreover, close to the keel of the last whorl there are one to two unspotted cords. Here on the bottom of the shell, the lateral cords are tighter and finer than those visible on the whorls.

Overall, this description matches the specimens described by March. Monterosato as *Jujubinus igneus* in the collection of the Hofmuseum (Imperial Museum of Natural History), therefore I chose this name. Only the livelier red colour (receding of the olive green colour and prevailing of yellow and pinkish) and the here bright red and yellow spotted, prominent sutural varix are important characters.

I would like to describe *Tr. igneus* Monteros. in coll. as a variety of *Tr. exasperatus* Penn., following the notion of “Les Mollusques marins du Roussillon” p.362–369 by Bucquoy, Dautzenberg and Dollfus. However, I have to remark that this species (*exasperatus*) falls within the considerable synonymy of *Trochus* (*Zizyphinus*) *exiguus* Pulteney (Kobelt, Prodr. p. 238; Carus, Prodr. p. 259).

Brusina and Stossich mention *Tr. exiguus* Pult. as *crenulatus* Brocchi for the Adriatic.

**Figure 2:**
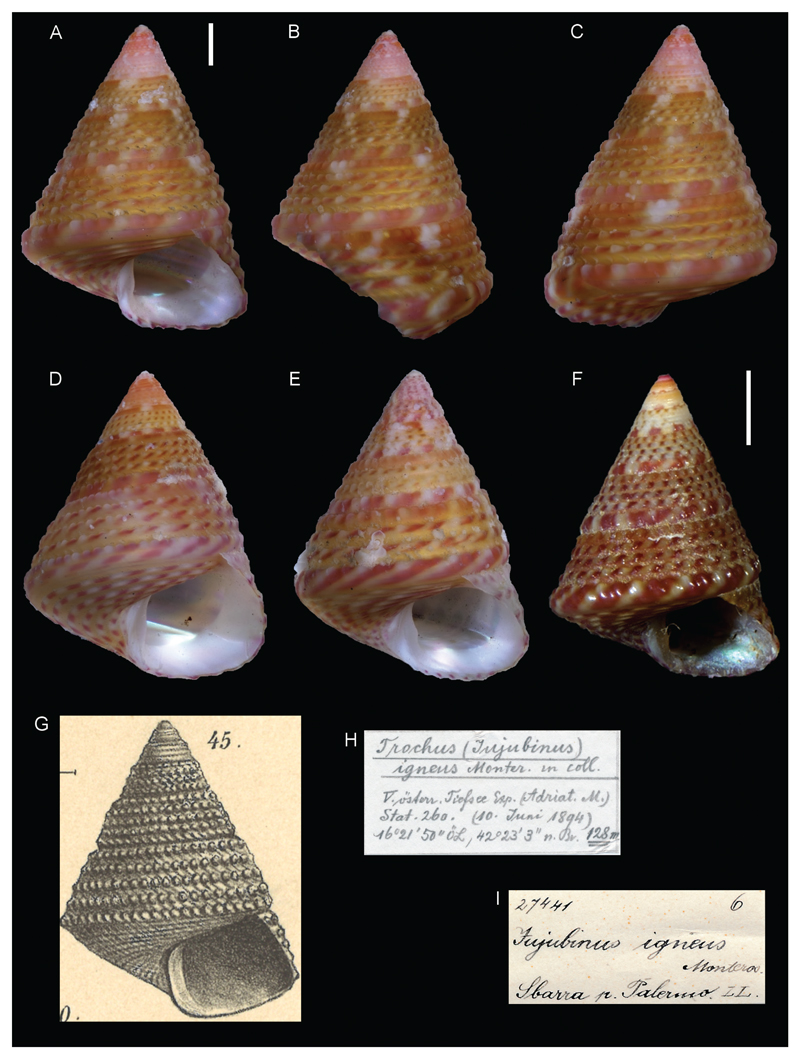
*Jujubinus igneus* Sturany, 1896 ex Monterosato ms. **A-C**. Syntype NHMW 72399a, station 260, Pelagruža, Croatia, 128 m: front (A), side (B) and back (C) views. **D–E**. Syntype NHMW 72399b and c: front views. **F**. NHMW 27441 (Monterosato coll.): front view. **G**. Original figure in Sturany, 1896. **H**. Original label of lot NHMW 72399. **I**. Original label of lot 27441. Scale bar: 1 mm.

**Comments.** Sturany identified these shells by comparison to specimens in the Monterosato collection (NHMW 27441) which closely match the specimens found in the “Pola” expedition (Fig. 2 A-F). However, Monterosato never formally introduced the name. Therefore, the variety *igneus* has to be considered a new subspecific name introduced by Sturany following the provisions of art. 45.6.4 of the ICZN code. This name is considered a junior synonym of *Jujubinus exasperatus* (Pennant, 1777) (MolluscaBase 2017). The name *Trochus* (*Jujubinus*) *igneus* was used also by Brusina (1896), whose work postdates Sturany’s one because he explicitly mentioned the preprint of Sturany’s work, and later by Locard (1904).

**Family Epitoniidae Berry, 1910** 

***Scalaria cerigottana* Sturany, 1896**

Fig. 3

**Figure 3:**
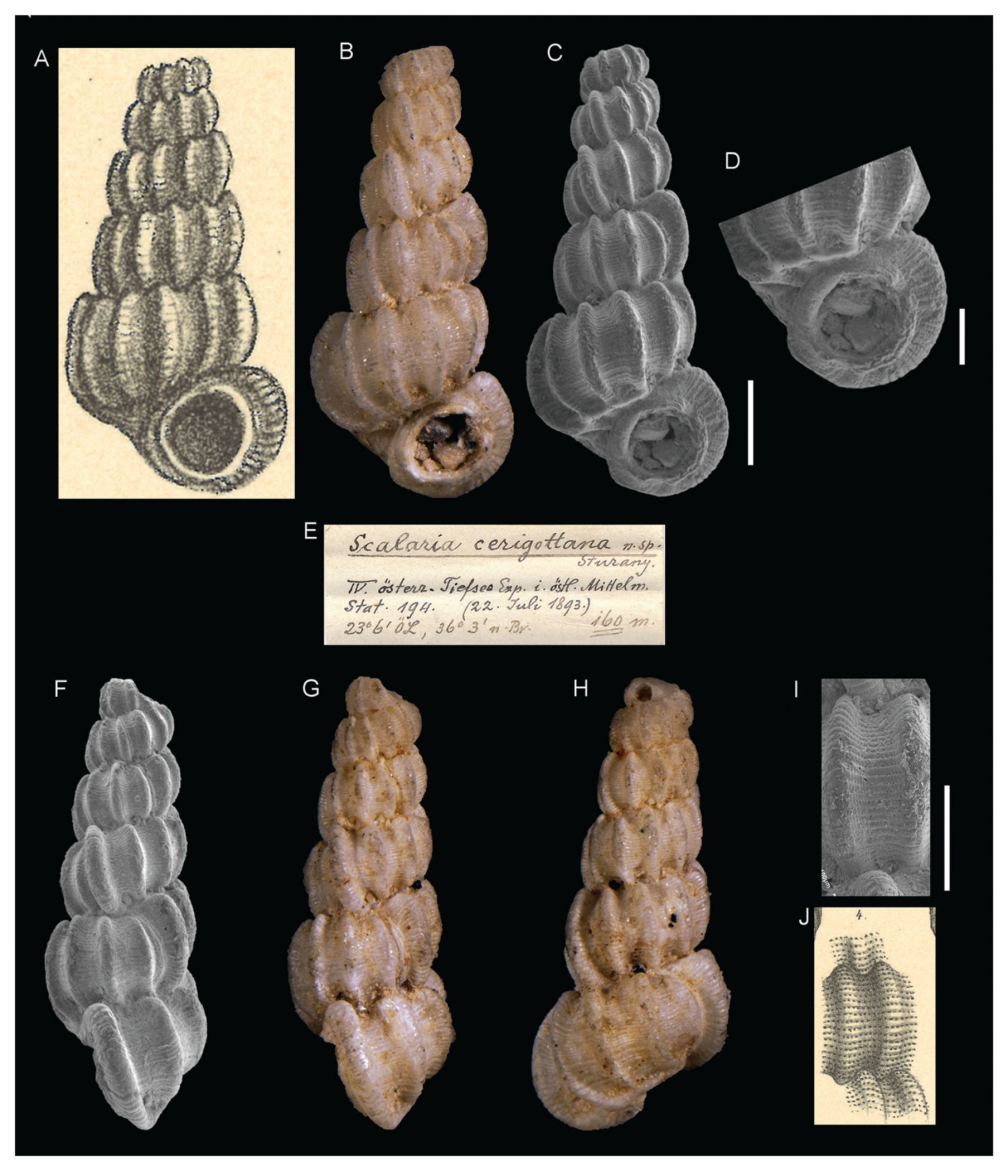
*Scalaria cerigottana* Sturany, 1896, Station 194, between Kythira and Antikythera, Ionian Sea, Greece, 160 m. **A**, **J**. Original figures in Sturany, 1896. **B–D, F–I**. Holotype NHMW 13001: front (**B–C**), side (**F–G**), and back (**H**) views, aperture (**D**), microsculpture (**I**). **E.** Original label. Scale bar: 1 mm (**B–C**, **F–H**); 0.5 mm (**D**, **I**).

**Figure 4:**
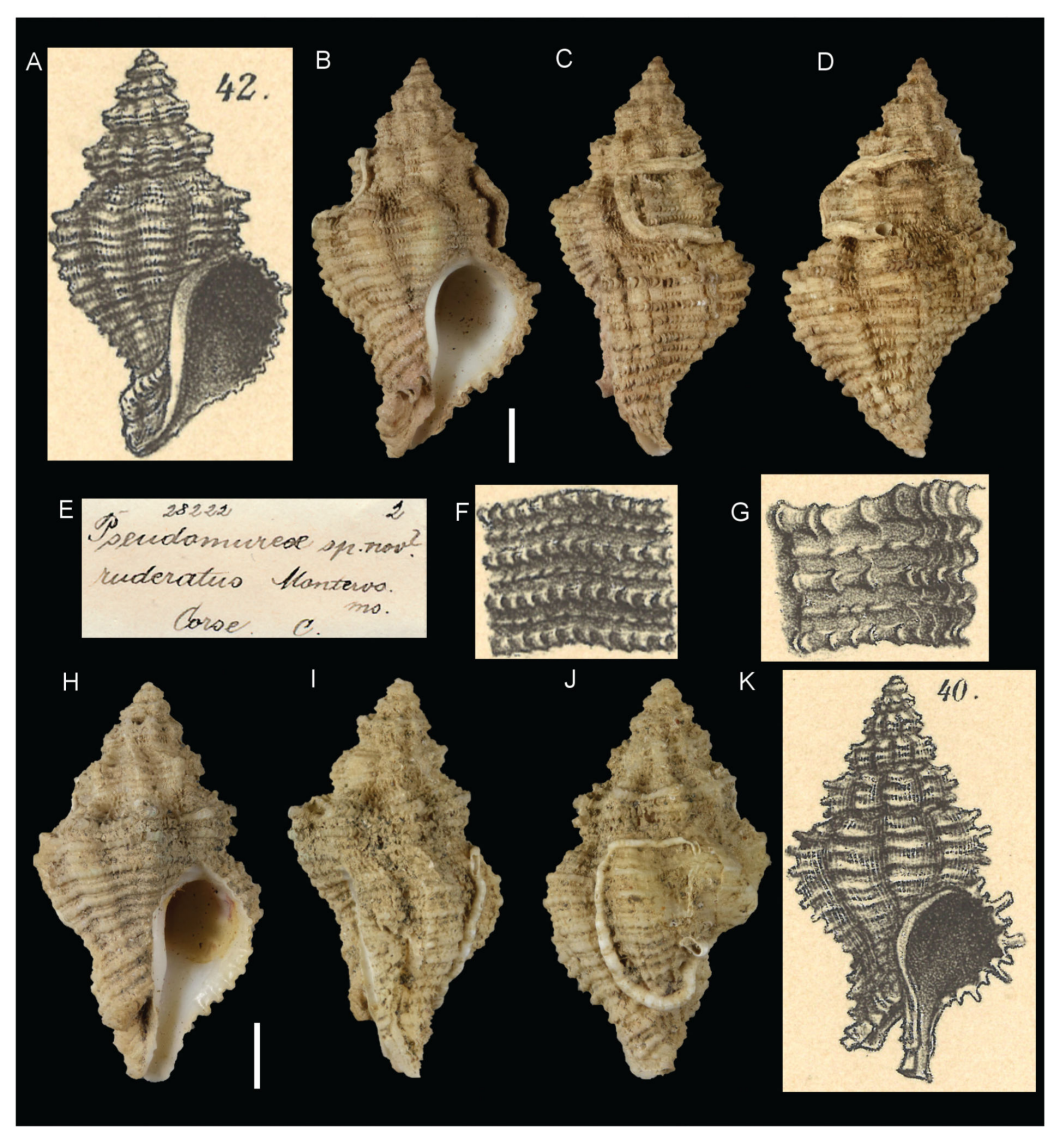
**A–F**, **H–J**. *Pseudomurex ruderatus* Sturany, 1896 in Monterosato ms., Corsica, France. Original figures in Sturany, 1896 (**A**, **I**). Specimen NHMW 28222a: front (**B**), side (**C**) and back (**D**) views, original label (**E**). Specimen NHMW 28222b: front (**H**), side (**I**) and back (J) view. **G**, **K**. *Fusus craticulatus* var. *pianosana* Sturany, 1896, Station 243, between Tremiti Islands and Pianosa Isl., Adriatic Sea, Italy, 103 m, original figures in Sturany, 1896. Scale bars: 1 mm.

Sturany 1896: 9, plate I, figures 3–4.

**Type locality.** Station 194, Kythira and Antikythira, Ionian Sea, Greece, 36°3'N, 23°6'E, 160 m.

**Type material.** Holotype: NHMW 13001, height 4.68 mm.

**Original description.** *Von Station 194 (160 m)*.

*Das einzige Exemplar dieser neuen Art besitzt nur 5½ Umgänge, indem der Apex des Gehäuses fehlt*.

*Auf jedem Umgange stehen 10 mächtige Querrippen. Diese sind aber nicht gleichmässig stark entwickelt, sondern es tritt hier und dort eine besonders dicke und hohe Rippe auffallend hervor, und gleich darauf folgt wieder eine solche unter dem Mittelmasse. Zwischen den einzelnen Rippen und über sie selbst hinweg ziehen zahlreiche (mehr als 20) Spiralstreifen, welche sich, mit dem Mikroskop betrachtet, als feine Furchen repräsentieren. (Fig. 4.) In diesen Furchen reihen sich kleine Grübchen aneinander, welche die Kreuzungspunkte von ursprünglich vorhandenen, bei unserem Exemplare aber nicht mehr sichtbaren Querfurchen mit den noch deutlich vorhandenen Spiralfurchen vorzustellen scheinen. (Man vergleiche diesbezüglich die mikroskopische Skulptur von* Scalaria funiculata *Watson.)*

*Die Rippen senken sich oben wie unten bogenförmig, also nicht winkelig, wie bei den nächstverwandten Arten, in die tiefe Naht; rings um den Nabel aber bilden sie durch wulstige Querverbindungen ihrer unteren Enden einen förmlichen Kiel. Dem kreisrunden Mundrand steht außen eine mächtige Rippe so nahe an, dass er wie verdoppelt aussieht*.

*Höhe des Gehäuses 5 mm, Breite 2,2 mm; Durchmesser der Mündung 1 mm*.

*Die Art ist am nächsten verwandt mit* Scalaria funiculata *Wats. von Pernambuco (Report on the scient. Res. of the Voyage of H.M.S. Challenger, Zool. Vol. XV,p. 141, pl. IX, fig. 4) und unterscheidet sich von dieser hauptsächlich durch das weniger zugespitzte Gehäuse und die ungleichmäßige Berippung. Ferner stehen ihr nahe* Scalaria longissima *Seg. (Kobelt Prodr. p. 77) und die fossilen Formen* Turbo torulosus *Brocchi (Conch. foss. subapp. 2. ed., vol. II, p. 163, pl. VII, fig. 4),* Scalaria plicosa *Phil. (Enum. Moll. Sicil. vol. II, p. 146, tab. XXIV, fig. 25),* Nodiscala cavata *de Boury (Bull. Soc. Mal. Ital. XIV, 1889, p. 173, tab. IV, fig. 13)*.

**Translation.** From station 194 (160 m).

The single specimen of this new species shows only 5 ½ whorls, as the apex is missing from the shell. Each whorl has 10 prominent axial ribs which are not developed equally, but some here and there are remarkably thick and high, surrounded by others smaller than average. In between the ribs and covering them are numerous (more than 20) spiral threads, which look like small grooves under the microscope (Fig. 4). Small pits ornament the threads. They seem to be crossing points of the spiral threads with axial lines which are no longer visible in our specimen (in this regard compare the microsculpture of *Scaria funiculata* Watson).

In contrast to closely related species, the ribs sink into the deep suture in a curved manner, not angled. However, they form a keel around the umbilicus through the thick interconnections of their lower ends. A thick varix is present on the lip and makes it seem duplicated.

The shell is 5 mm high, 2.2 mm wide and the diameter of the mouth is 1 mm.

The most closely related species is *Scalaria funiculata* Wats. of Pernambuco (Report on the scient. Res. of the Voyage of H.M.S. Challenger, Zool.Vol.XV, p. 141, pl. IX, fig. 4) and can be distinguished from it by the less pointed shell and the uneven rib pattern. Other closely related species are *Scalaria longissima* Seg. (Kobelt Prodr. p.77) and the fossil forms *Turbo torulosus* Brocchi (Conch. foss. subapp. 2. ed., vol. II, p. 163, pl. VII, fig. 4), *Scalaria plicosa* Phil. (Enum. Moll. Sicil. vol. II, p. 146, tab. XXIV, fig. 25), *Nodiscala cavata* de Boury (Bull. Soc. Mal. Ital. XIV, 1889, p. 173, tab. IV, fig. 13).

**Comments.** A valid taxon accepted as *Punctiscala cerigottana* (MolluscaBase 2017).

**Family Muricidae Rafinesque, 1815** 

***Fusus craticulatus* var. *pianosana* Sturany, 1896**

Fig. 4G, K

Sturany 1896: 25-26, plate II, figures 40–41.

**Original locality.** Station 243, between Tremiti Islands and Pianosa Isl., Adriatic Sea, Italy, 42°11'40"N, 15°40'50"E, 103 m.

**Type material.** not found.

**Original description.** *Von Station 243 (103 m); 1 Exemplar*.

*Das 31 ½ mm hohe und 16 mm breite Gehäuse ist eine Missbildung, indem der Stiel doppelt ausgebildet wurde*.

*Der ursprünglich angesetzte Stiel steht links ab und bildet einen tiefen nabelartigen Trichter, während ihn rechts der neugebildete an Länge überragt. Die Mündung beträgt zusammen mit dem Stiele, der, nebenbei bemerkt, nirgends mit seinen Rändern verwachsen, sondern durchgehend offen rinnenförmig ist, 15 ½ mm in der Länge. Vom Apex ist ein kleines Stück abgebrochen, 7 Windungen sind erhalten*.

*Die Sculptur der Umgänge erinnert an die der Coralliophilen. Eine Anzahl von mit Schuppen mehr minder reich besetzten Spiralreifen läuft über sie hinweg; an den oberen Umgängen sind 3 bis 5 solcher Reifen vorhanden, und zwar sind sie ziemlich gleich, stark (breit) und noch wenig beschuppt; an den letzten zwei Windungen treffen wir schon bedeutend mehr, und hier wechseln stärkere (breite) und schwächere (schmale) ziemlich regelmäßig ab, d. h. zwischen zwei starke oder Hauptreifen erscheint meistens ein ganz zarter, aber deshalb nicht schuppenloser Reifen eingeschoben. Querwülste sind auf den letzten Wildungen 8–9 vorhanden, oben etwas weniger. Der Aussenrand der Mündung ist entsprechend den dort endigenden Spiralreifen gezackt*.

*Für die* Coralliophila squamulosa *Phil. (Kobelt, Prodr. p. 15; Carus, Prodr. p. 380) ist das Exemplar erstens zu gross, und zweitens hat es nur 9 Querfalten auf dem letzten Umgange, während jene Art 12 bis 13 haben soll. Vielleicht aber sind Herrn March. Monterosato ähnliche Formen vorgelegen, als er sich entschloss, die* Coralliophila squamulosa *als Varietät von* Murex brocchi*, resp*. Fusus craticulatus *zu erklären (Monterosato, Nuova Rivista, p. 39)*.

*In der Monterosato-Collection des naturhistorischen Hofmuseums in Wien sind zwei sehr interessante, der hier in Rede stehenden Form nahe kommende Exemplare mit der Determination „*Pseudomurex sp. nov*.? (*ruderatus *Monts ms.)“ aufbewahrt. Sie sind blos durch eine viel größere Anzahl von Spiralreifen, welche überdies auch viel gleichmässiger stark sind, zu unterscheiden, sowie durch die relativ minder schlanke Form (Höhe 26 ½, Breite 15 mm.). Ich habe eines dieser Exemplare zum Vergleiche abgebildet (Taf. II, Fig. 42 und 43)*.

*Von dem echten* Fusus craticulatus *Brocchi (=*Hadriana brocchi *Mont.), zu dem ich unser Exemplar vorläufig als Varietät stellen muss, ist dieses aber ebenfalls durch die bedeutend stärker beschuppten Reifen und die geringere Anzahl derselben unterschieden*.

**Translation.** From station 243 (103 m); 1 specimen.

The shell is 31 ½ mm high and 16 mm wide. A deformation led to doubled development of the siphonal canal. The original siphonal canal is pointed towards the left and forms a deep, umbilicus-resembling funnel, while it is overtopped in length by the secondary siphonal canal to the right.

The mouth opens into the siphonal canal, whose margins never touch each other, is continuously open and 15 ½ mm long. The apex is slightly damaged, seven whorls are preserved.

The sculpture of the whorls resembles those of the coralliophilas; it is accompanied by a number of spiral cords covered unevenly with scales. On the top whorls, there are 3 to 5 such formations which are even and strong (broad) and show only few scales. On the last two whorls, we find considerably more, which alternate quite regularly between stronger (broader) and weaker (narrower) formations. One narrow, scale covered cord is periodically found after two strong spiral stripes. On the last whorls there are varices which are slightly less prominent on the top whorls. The lip is toothed, corresponding to the spiral stripes ending there.

The specimen is too large to be *Coralliophila squamulosa* Phil. (Kobelt, Prodr. p. 15; Carus, Prodr. p. 380) and it has only nine lateral folds on the last whorl, while that species supposedly has 12 to 13. Perhaps though, Mr. March. Monterosato was looking at similar forms when he decided to attribute

*Coralliophila squamulosa* as a variety of *Murex brocchi,* resp*. Fusus craticulatus* (Monterosato, Nuova Rivista, p. 39).

There are two very interesting specimens stored in the Monterosato collection of the Imperial Museum of Natural History, which resemble the available specimen and are determined as “*Pseudomurex sp. nov*.? (*ruderatus* Monts ms.)“. They can be distinguished merely due to a much larger number of spiral stripes, which are also more even in thickness, as well as a relatively less slender shape (height 26 ½ mm, width 15 mm). I have depicted one of these specimens as a comparison (Plate II, Fig. 42 and 43).

However, it is distinguished from the real *Fusus craticulatus* Brocchi *(=Hadriana brocchi* Mont.) by stronger scale coverage on the spiral stripes and a smaller number of those very stripes.

**Comments.** Sturany introduced the name *pianosana* for a variety of *Fusus craticulatus* Brocchi, 1814 found during the “Pola” expeditions; it has a subspecific rank according to the art. 45.6.4 of the ICZN Code. This name is currently considered a junior synonym of *Hirtomurex squamosus* (Bivona Ant. in Bivona And., 1838) (MolluscaBase 2017).

***Pseudomurex ruderatus* Sturany, 1896 in Monterosato ms**.

Fig. 4A–F, H–J

Sturany 1896: 26, plate II, figures 42–43.

**Original locality.** Corsica, France (from label accompanying the type material).

**Type material.** NHMW 28222: 2 syntypes, Corsica, France (Monterosato coll.).

**Original description and translation.** see *Fusus craticulatus* var. *pianosana*.

**Comments.** Sturany introduced the name *Pseudomurex ruderatus* on the basis of two shells and a label of the Monterosato collection. He stated the main differences from similar species, illustrated the shells and rendered the name available. This name is currently considered a junior synonym of *Hirtomurex squamosus* (Bivona Ant. in Bivona And., 1838) (MolluscaBase 2017).

**Family Fasciolariidae Gray, 1853** 

***Fusus bengasiensis* Sturany, 1896**

Fig. 5

Sturany 1896: 8, plate I, figures 1–2.

**Type locality.** Station 36, north of Benghazi, Libya, 32°46'40"N, 19°58'30"E, 680 m.

**Type material.** Holotype: NHMW 13000, height 45.23 mm.

**Original description.** *Von Station 36 (680 m); subfossil*.

*1 Exemplar von 45 ½ m Länge und 18 ½ mm grösster Breite; die Länge der Mündung beträgt 28 mm, wovon etwa 15 mm auf den oben offenen Stiel kommen, die Breite derselben 9 mm*.

*Die Spitze des Gehäuses fehlt und nur mehr 6 Windungen sind erhalten. Ein besonders an den untersten Windungen stark hervortretender Kiel verläuft etwas unter der Mitte und bildet überall da, wo er die spärlich vertretenen Querwülste durchkreuzt, von oben nach unten abgeflachte Fortsätze. Die Anzahl derselben richtet sich also nach derjenigen der Querwülste, dies sind 6 auf der letzten Windung und 8–9 auf den übrigen. Oberund unterhalb des Kieles verlaufen in grösserer Anzahl Spiralreifen, ganz wie bei* Fusus rostratus *in der Dicke (Stärke) etwas variabel, aber nicht geschuppt, sondern nur verwischt quergestreift. Die Naht schneidet tief ein. Der Innenrand der Mündung ist über dem Spindelrande in einer zur letzten Windung senkrecht stehenden, wellenförmig geschwungenen Platte losgelöst. Der Stiel ist ein wenig um seine Axe gedreht*.

*Auf den ersten Blick möchte man die eben beschriebene Form wohl für eine Varietät von* Fusus rostratus *Oliv. halten, doch wäre diese Deutung im Hinblicke auf den unter die Mitte gerückten, exorbitant scharf vortretenden Kiel nicht gerechtfertigt*.

**Translation.** From station 36 (680 m); subfossil.

One specimen of 45.5 mm length and 18.5 mm greatest width; the mouth is 9 mm wide and 28 mm long, 15 of which belong to the siphonal canal which is open on top. The apex of the shell is missing and only 6 whorls are preserved. A keel which is especially prominent on the lowest whorls runs in the lower half of the whorl and forms two flattened protuberances wherever it crosses the sparse axial ribs. Therefore, the number of these protuberances corresponds to the number of axial ribs, which is 6 at the body whorl and 8–9 at the spire whorls. A greater number of spiral cords run above and below the keel. They vary slightly in thickness, resembling those in *Fusus rostratus*, but without scales and are only poorly transversely ribbed. The suture is deep. The inside of the aperture is loosened above the columellar lip in an undulated callus which is perpendicular to the last whorl. The siphonal canal is bent.

At first appearance, the form described here could be mistaken for a variety of *Fusus rostratus* Oliv., however, this interpretation would not be justified, due to the centred, exorbitantly sharp protruding keel.

**Comments.** *F. bengasiensis* is considered a junior synonym of *Fusinus rostratus* (Olivi, 1792) (Buzzurro and Russo 2007).

**Family Marginellidae Fleming, 1828** 

***Marginella occulta* var. *minor*** **Sturany, 1896**

Fig. 6

Sturany 1896: 9, not illustrated.

**Examined material.** NHMW 13012: 2 specimens, station 194. NHMW 99998: 1 specimen, station 36.

**Original description.** *Von den Stationen 36 (680 m) und 194 (160 m)*.

*Es liegen von Station 194 zwei Exemplare von 2,3 mm Höhe und 1,7 mm Breite vor, von Station 36 ein größeres mit den Massen 3,1: 2,1 mm*. *Nur letzteres stimmt mit dem Typus der Art überein, der sich in der Monterosato-Collection des naturhistorischen Hofmuseums befindet, und den dasselbe nur noch in der Grösse übertrifft. (Exemplare von March. Monterosato messen 2,7 mm in der Höhe und 1,7 mm in der Breite.)*

*Die kleinen Stücke von Station 194 aber dürften zu der bisher nur in coll. bekannt gewordenen* M. (Gibberulina) obtusa *Monter. gehören, mit der sie sich in Form und Ausmass ziemlich decken. (Exemplare von Monterosato sind 2,1 – 2,2 mm hoch und 1,6 – 1,7 mm breit.) Doch erscheint es mir nicht empfehlenswerth, eine Species-Trennung vorzunehmen, da die* M. (Gibberulina) obtusa *Monter. schwerlich etwas anderes als eine kleine Varietät der* M. occulta *Monter. ist. (etwa* M. occulta *Monter*. var. minor = M. (Gibberulina) obtusa *Monter. in coll*.).

**Translation.** From stations 36 (680 m) and 194 (160 m).

There are two specimens (2.3 mm height and 1.7 mm width) available from station 194. A larger specimen (3.1 : 2.1 mm) is available from station 36. Only the latter matches the type specimen in the Monterosato-Collection of the k.k. Naturhistorisches Hofmuseum (Imperial Museum of Natural History) which is merely surpassed in size (specimens of March. Monterosato are 2.7 mm high and 1.7 mm wide).

The small specimens from station 194, however, should belong to *M. (Gibberulina) obtusa* Monter., only known *in coll*., which is rather congruent in form and size (specimens from Monterosato measure 2.1 – 2.2 mm in height and 1.6 – 1.7 mm in width). Still, I do not consider recommendable to separate the species, because *M. (Gibberulina) obtusa* Monter. is hardly anything but a smaller variety of *M. occulta* Monter. (*M. occulta* Monter. var. *minor* = *M. (Gibberulina) obtusa* Monter. in coll.).

**Comments.** Sturany identified the small specimens from station 194 as *Marginella obtusa* Monterosato, 1878, considered a *nomen nudum* by Gofas (1992). Sturany clearly stated that he considered this species *schwerlich etwas anderes als eine kleine Varietät der* M. occulta [hardly anything but a smaller variety of *M. occulta*] and that *doch erscheint es mir nicht empfehlenswerth, eine Species-Trennung vorzunehmen* [I do not consider recommendable to separate the species]. He used the “var. *minor*” statement only to highlight the small size of these specimens. Such a statement implies that the name is not available (art. 11.5 of the ICZN code).

**Family Raphitomidae Bellardi, 1875** 

*Defrancia implicisculpta* Sturany, 1896

Fig. 7

Sturany 1896: 12, plate I, figure 10-12.

**Type locality.** Station 82, north of Alexandria, Egypt, 32°30'N, 29°8'E, 2420 m.

**Type material.** Holotype: NHMW 13003, height 3.39 mm.

**Original description.** *von Station 82 (2420 m); 1 Exemplar*.

*Das Gehäuse ist spindelförmig, von graubrauner Farbe und besitzt 6 ½, Umgänge, wovon beinahe 4 auf das sogenannte Embryonalgewinde entfallen. Dieses zeigt, allerdings nur stellenweise, ein fein gegittertes Netzwerk (Fig. 12), während auf den 2 ½ unteren Windungen eine hiervon ganz verschiedene Sculptur (Fig. 11) auftritt. Unterhalb der Naht nämlich liegt ein schmaler, concaver Theil mit vielen bogenförmigen Querstrichen, und auf diesen folgt, durch 1 oder 2 Spirallinien gleichsam abgetrennt, der übrige Theil der Umgangsbreite. Dieser ist convex und trägt derbe Querrippen oder Querwülste (10 auf dem vorletzten, 12 auf dem letzten Umgange), durchkreuzt von ziemlich starken Spiralreifen (3 auf dem vorletzten und 6 auf dem letzten Umgange). Die Mündung ist birnförmig, hat oben am Aussenrande einen kleinen Ausschnitt und ist unten in den Stiel ausgezogen, um welchen ebenfalls einige Spiralreifen verlaufen*.

*Höhe des Gehäuses 3,5, Breite desselben 2,0 mm; Höhe der Mündung samt Stiel 2 mm*.

*In der Gestalt ist diese neue Art ähnlich der* Defrancia cordieri *Payr. (Kobelt Prodr, p. 143) und deren verwandten Formen, für welche Monterosato die Gattung* Cordieria *aufstellt, und auch mit einer noch nicht publizierten Art, mit* Cordiera hispida *Mont. in coll., welche mir aus der Sammlung des Hofmuseums zum Vergleiche vorliegt, zeigt sie vielfach Ähnlichkeit, doch scheint sie schon durch ihre geringen Dimensionen genügend charakterisiert und unterschieden zu sein*.

**Translation.** From station 82 (2420 m); 1 specimen.

The gray brown shell is fusiform and has 6 ½ whorls, almost 4 of which belonging to the so-called protoconch. This shows, in some parts, a finely reticulated sculpture (Fig. 12), while on the lower 2 ½ whorls there is a completely different sculpture (Fig. 11). Below the suture lies a slender, concave part with many arched axial lines, which is followed by the remaining part of the whorl’s width, separated by 1 or 2 spiral lines. This remaining part is convex and is provided with solid axial ribs (10 on the penultimate, 12 on the last whorl), crossed by rather strong spiral stripes (3 on the penultimate, 6 on the last whorl). The aperture is pear-shaped with a small opening on the upper outer edge and is elongated downwards and blended into the siphonal canal, which is also ornamented by some spiral stripes.

The length of the shell is 3.5 mm, the width 2 mm, and the height of the aperture with the siphonal canal 2 mm.

In its form, this new species resembles *Defrancia cordieri* Payr. (Kobelt Prodr, p. 143), as well as its related forms for which Monterosato establishes the genus of *Cordiera*. It also shows multiple similarities to an unpublished species to date, *Cordiera hispida* Mont. in coll., which is available to me as an object of comparison from the museum collection. However, it seems that the characterization and distinction can be made sufficiently due to its smaller dimensions.

**Comments.** Currently considered a junior synonym of *Pleurotomella eurybrocha* (Dautzenberg & Fischer, 1896) (Bouchet and Warén 1980).

**Raphitoma *nuperrima*** var. **corallinoides** Sturany, 1896 and var. *pseudacanthodes* Sturany, 1896

Fig. 8

Sturany 1896: 10-11, plate I, figure 6 (var. *pseudacanthodes*) and 7 (var. **corallinoides**).

**Type locality.** var. **corallinoides**: Station 36, north of Benghazi, Libya, 680 m. var. *pseudacanthodes*: Station 1, west of Corfu, Greece, 615 m; Station 213, north of Astypalaia, Dodekanese, Greece, 597 m.

**Type material.** var. **corallinoides**: Holotype: NHMW 13013, height 7.27 mm; var. *pseudacanthodes*: NHMW 13014: 1 syntype, station 1; NHMW 13015: 9 syntypes, station 213.

**Original description.** *Von den Stationen 1, 36, 213 (597–680 m), und zwar von den ersteren je 1 Exemplar, von der letzteren eine grössere Anzahl*.

*Auf die Beschreibungen, welche von dieser Art in der Literatur zu finden sind, passen die Exemplare der österreichischen Tiefsee-Expeditionen recht gut, mit den bis jetzt vorhandenen Abbildungen harmonieren sie weniger*.

*Es soll gezeigt werden, dass nicht alle der vorliegenden Exemplare dem Typus angehören, und deshalb ist es zunächst notwendig, einige Beispiele über Massverhältnisse anzuführen*.

[A table with the sizes of the collected specimens follows]

**Figure 5:**
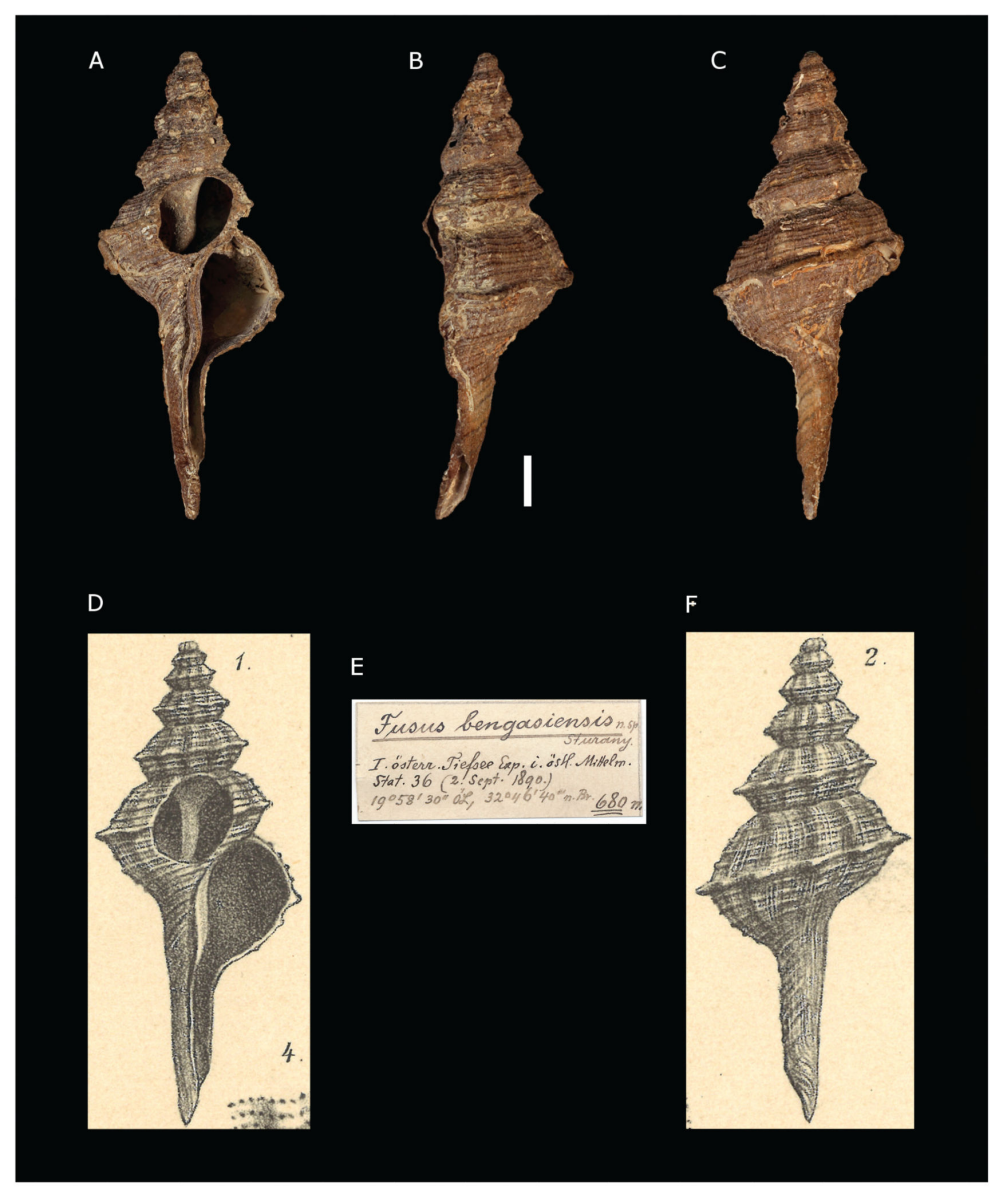
*Fusus bengasiensis* Sturany, 1896, Station 36, north of Benghazi, Libya. **A–C**. Holotype NHMW 13000: front (**A**), side (**B**), and back (**C**) views **E**. Original label. **D**, **F**. Original figures in Sturany, 1896. Scale bar: 5 mm.

**Figure 6:**
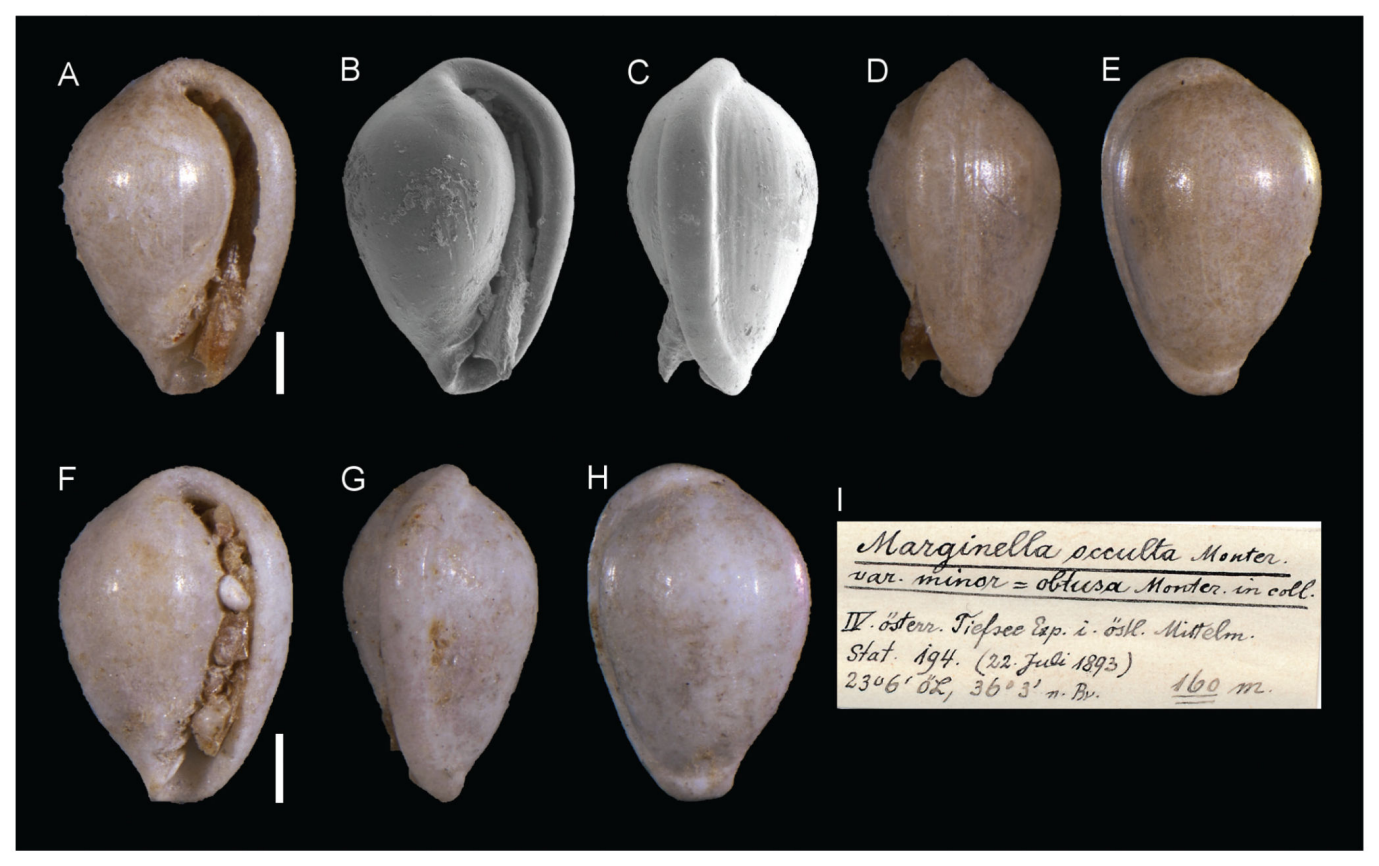
*Marginella occulta* var. *minor* Sturany, 1896, Station 194, between Kythira and Antikythira, Ionian Sea, Greece, 160 m. **A–E**. NHMW 13012: front (**A–B**), side (**C–D**) and back (**E**) views. **F–H**. NHMW 13012: front (**F**), side (**G**) and back (**H**) views. **I**. Original label. Scale bars: 0.5 mm.

*Also nur 1 Exemplar erreicht annähernd die in der Literatur als typisch angegebene Höhe von 12 mm, alle übrigen sind von weit kleineren Dimensionen. Ferner zeigen sie mit Ausnahme von 2 Exemplaren, auf die ich unten als Typen noch zurückkomme, alle eine nicht blos absolut, sondern auch relativ geringe Gehäusebreite als der Typus, der mir in einem leider zerbrochenen ausgewachsenen und einem unfertigen Exemplare aus der Monterosato-Sammlung zum Vergleiche vorliegt. Dieser Typus weist auch eine viel grössere Anzahl von Spiralrippen und Spiralstreifen, welche auf allen Windungen markant hervortreten, auf, während bei unseren Exemplaren die Rippen von der vorletzten Windung bis hinauf zum Embryonalgewinde nur in der Zweizahl vorhanden sind und eine schwache Kante bilden, wie dies bei* Pleurotoma (Mangelia) acanthodes *Wats. der Fall ist. Mit dieser von den Bermudas-Inseln und den Azoren stammenden Art haben unsere Exemplare auch die Dimensionen vollständig übereinstimmend, ferner die Skulptur des Embryonalgewindes, und streng genommen besteht eine Verschiedenheit unserer Exemplare von* acanthodes *Wats. eigentlich nur in ihrer reichlicheren Körnchensculptur, d. h. zwischen den Spiralrippen der letzten Windung stehen weit mehr Reihen von Punkten oder Körnchen als bei* acanthodes*, und ober der ersten Rippe des letzten Umganges, also zwischen ihr und der Naht, 10 bis 12 solcher Reihen. Wegen dieser grossen Ähnlichkeit der Mehrzahl der gesammelten Exemplare mit der Watson‘schen Art möchte ich sie von* *Raphitoma *nuperrima** Tib*. als* nov. var. pseudacanthodes *trennen, und dazu das grosse Exemplar von Station 1 (Fig. 6), sowie von Station 213 alle mit Ausnahme jener zwei Exemplare rechnen, welche ich gleich bei der Besprechung der Gehäusebreite ausgenommen habe. Diese muss ich, da sie, wie gesagt, relativ breiter sind, und da sie ferner mehr Spiralrippen besitzen (nämlich circa eben so viel wie die echte* *nuperrima* *in der Monterosato-Collection des Hofmuseums) als* *Raphitoma *nuperrima** Tib. typ. *isolieren (Fig. 5). Leider sind sie nicht erwachsen*.

**Figure 7:**
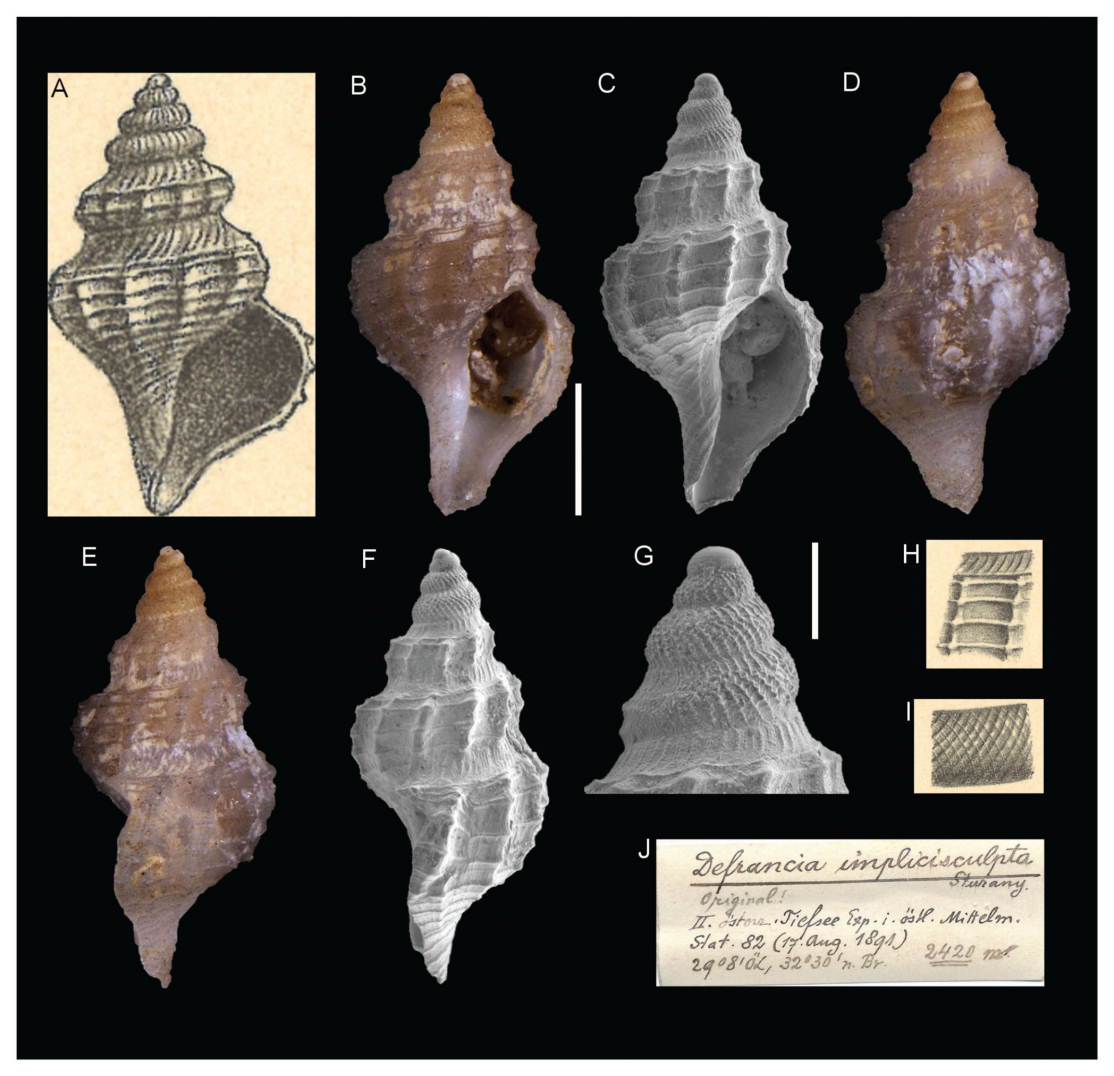
*Defrancia implicisculpta,* Sturany, 1896, station 82, north of Alexandria, Egypt, 2420 m. **A**, **H**, **I**. Original figures in Sturany, 1896. **B–G**. Holotype NHMW 13003: front (**B–C**), back (**D**), side (**E**–**F**) views and microsculpture (**G**). **J**. Original label. Scale bars: 1 mm (**B–F**), 0.3 mm (**G**).

*Schliesslich ist aber auch das Exemplar von Station 36, welches sich dadurch auszeichnet, dass zwischen den Spiralrippen 1 und 2 auf dem letzten Umgang ein grösserer Zwischenraum ist, als eine besondere Varietät zu bezeichnen, und zwar mit einer zweiten Watson’schen Art, mit* Pleurotoma (Mangelia) corallina. *von Westindien zu vergleichen, weshalb ich sie als* nov. var. *corallinoides* *aufführe (Fig. 7)*.

**Translation.** From stations 1, 36, 213 (597–680 m), one specimen from each of the first two stations and a larger number from the latter station.

The specimens of the Austrian deep-sea expedition fit the descriptions currently found in the literature rather well; however, they poorly match the available figures.

It shall be demonstrated that not all of the presented specimens belong to the type, and it is therefore necessary to give some information on the dimensions. Only one specimen reaches the size of 12 mm described in the literature as typical height, all the rest are of far smaller dimensions. Furthermore, with the exception of two specimens, they show a smaller shell not only in absolute terms but also in relation to the type which is available to me as an object of comparison, unfortunately in the form of a broken and juvenile specimen from the Monterosato collection. This type also shows a greater number of prominent spiral ribs and stripes on all whorls, while our specimens show only two ribs from the penultimate whorl upwards to the protoconch, which forms a slight edge, much as it is the case in *Pleurotoma (Mangelia) acanthodes* Wats. Our specimens also correspond completely with this species from the Bermuda Islands and Azores in dimensions, sculpture of the protoconch. Strictly speaking, there is no difference between our specimens and *acanthodes* Wats., except for its richer tubercled sculpture, i.e., between the spiral cords of the last whorl, there are much more rows of granulose structures than there are in *acanthodes*, and above the last rib of the last whorl, namely between it and the suture, there are 10 to 12 such cords. Because of that great similarity of the majority of the collected specimens with Watson’s species, I would like to separate it from **Raphitoma *nuperrima*** Tib. as nov. var. *pseudacanthodes* and include the large specimen from Station 1 (Fig. 6), as well as all those retrieved from station 213 with the exemption of those 2 specimens which I excluded from the review of shell sizes. As mentioned above, I have to isolate them as **Raphitoma *nuperrima*** Tib. *typ*. (Fig. 5), because they are relatively broader and possess more spiral ribs (approximately as many as the real **nuperrima** in the Monterosato collection of the Imperial Museum). Unfortunately, they are juveniles.

Finally, also the specimen retrieved from station 36 must be designated a remarkable variety. It distinguishes itself by a greater gap between spiral ribs 1 and 2 on the last whorl and must be compared to a second species by Watson, *Pleurotoma (Mangelia) corallina* from the West Indies, which is why I mention it as *nov. var. *corallinoides** (Fig. 7).

**Comments.** Both taxa were considered junior synonyms of *Bela *nuperrima** (Tiberi, 1855) (Bouchet and Warén 1980, MolluscaBase 2017) but more likely belong to *Kurziella serga* (Dall, 1881) (S. Gofas, pers. comm. November 2017).

*Taranis alexandrina* Sturany, 1896

Fig. 9

**Figure 8:**
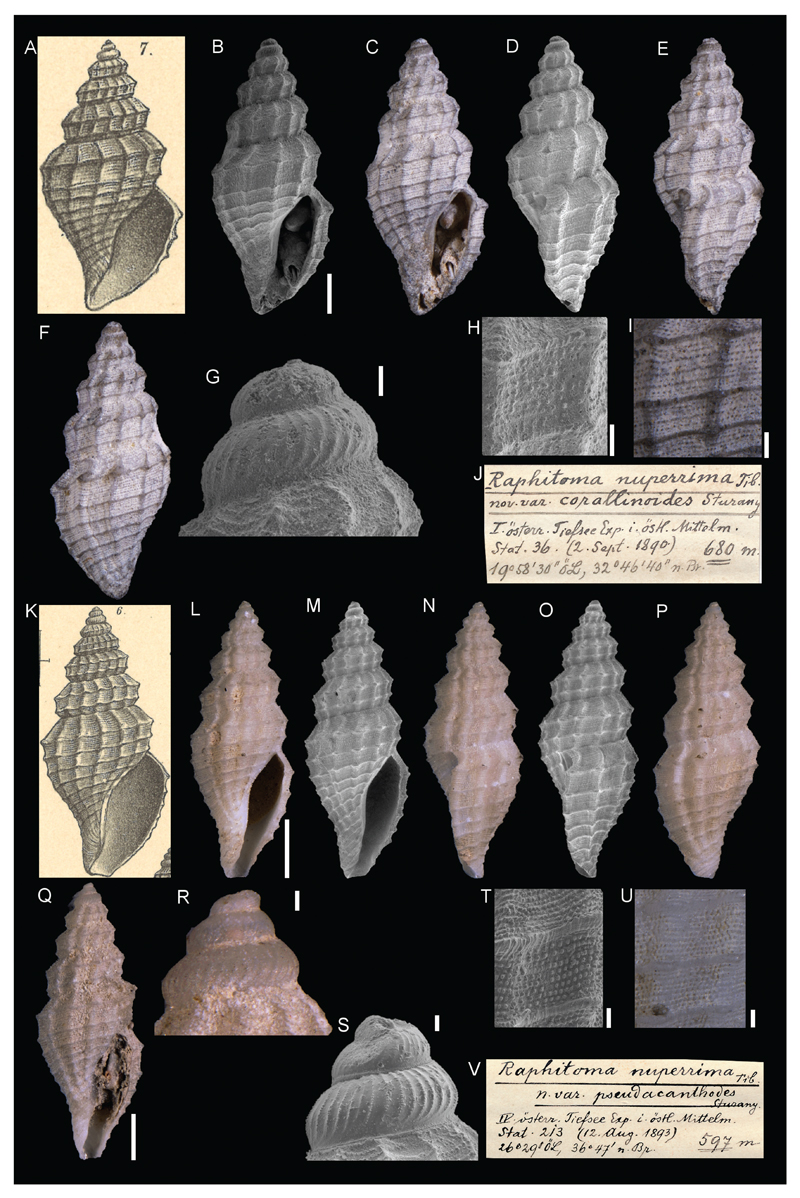
**A–J**. *Raphitoma nuperrima* var. *corallinoides* Sturany, 1896, holotype NHMW 13013, Station 36, north of Benghazi, Libya, 680 m: original figure in Sturany, 1896 (**A**), front (**B–C**), side (**D–E**) and back (**F**) views, apex (**G**), microsculpture (**H–I**), original label (**J**). **K–P**, **T–V**. **Raphitoma *nuperrima*** var. *pseudacanthodes* Sturany, 1896, syntype NHMW 13014a, Station 213, north of Astypalaia, Sporades, Greece, 597 m: original figure in Sturany, 1896 (**K**), front (**L–M**), side (**N–O**) and back (**P**) views, microsculpture (**T–U**), original label (**V**). **Q–R**. **Raphitoma *nuperrima*** var. *pseudacanthodes* Sturany, 1896, syntype NHMW 13014b (same locality as 13014a): front (**Q**), apex (**R–S**). Scale bars: 1 mm (**B–F**, **L–Q**); 0.1 mm (**G**, **R–U**); 0.2 mm (**H–I**).

**Figure 9:**
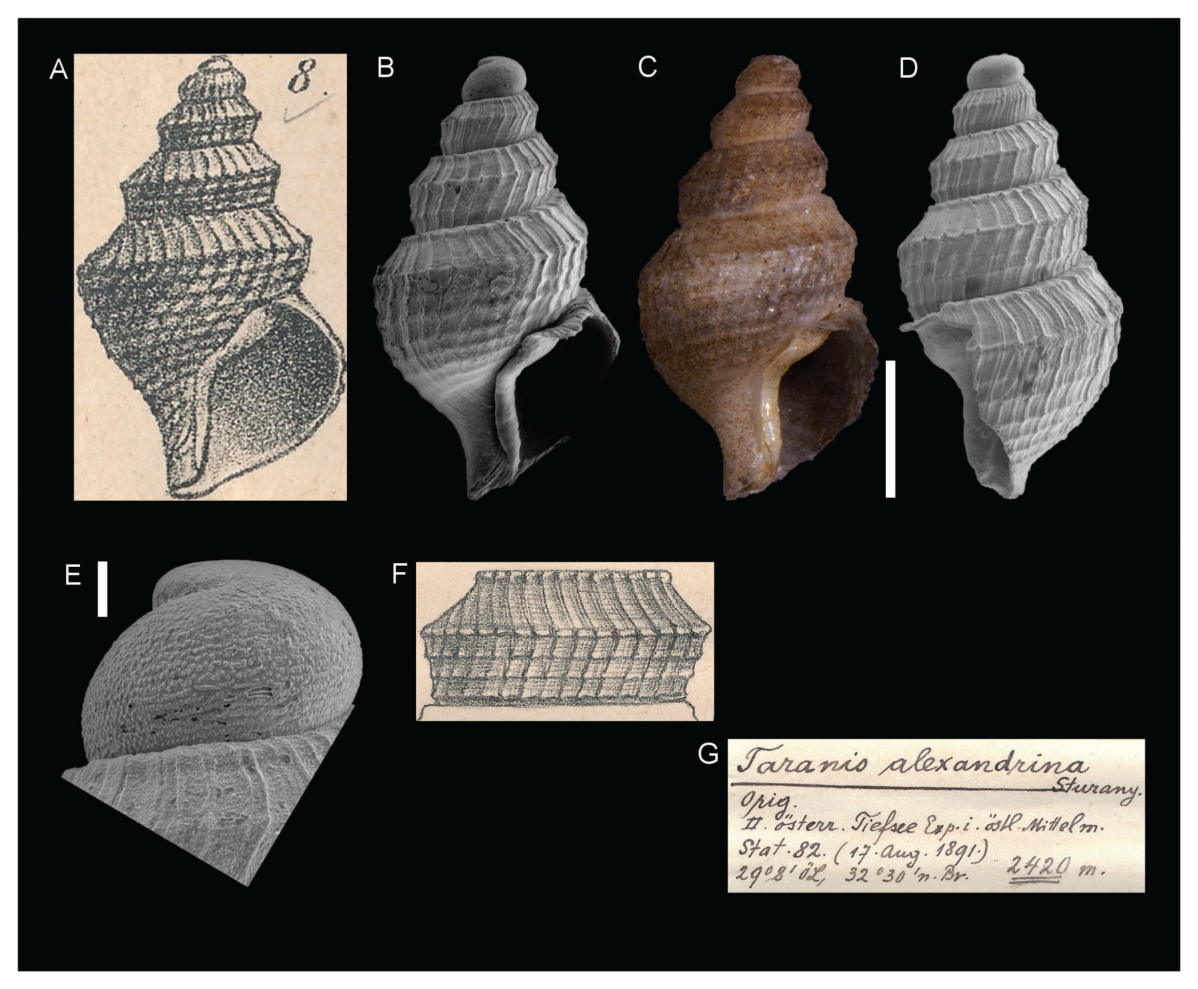
*Taranis alexandrina,* Sturany, 1896, station 82, north of Alexandria, Egypt, 2420 m. **A–F**. Holotype NHMW 13002: original figures in Sturany, 1896 (**A**, **F**), front (**B–C**) and side (**D**) views, apex (**E**). **G**. Original label. Scale bars: 1 mm (**B–D**); 0.1 mm (**E**).

**Figure 10:**
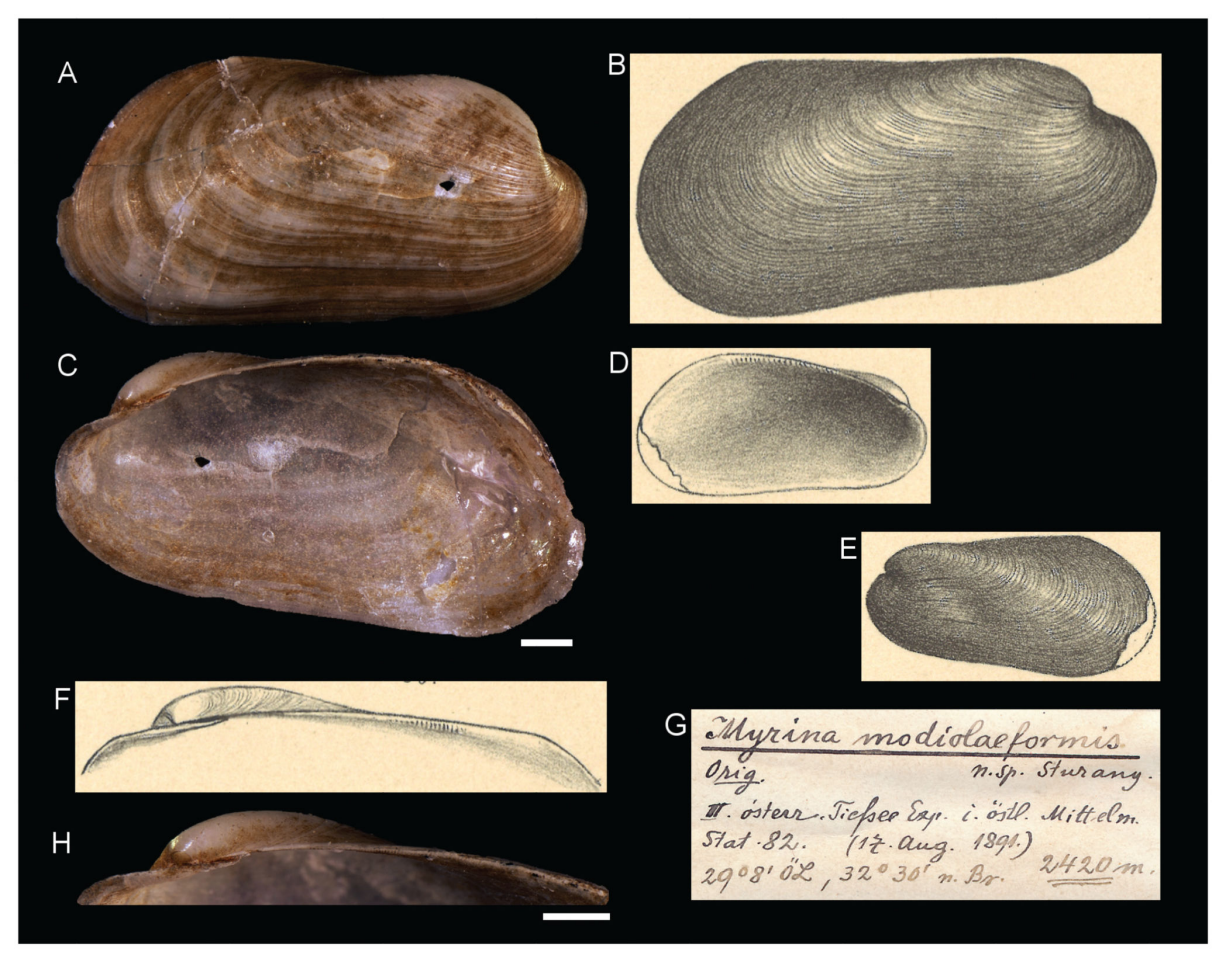
*Myrina modiolaeformis* Sturany, 1896, station 82, north of Alexandria, Egypt, 2420 m. **A**, **C**, **H**. Syntype NHMW 13011a: outer (**A**) and inner (**C**) view, hinge (**H**). **B**, **D**–**F**. Original figures in Sturany, 1896. **G**. Original label. Scale bars: 1 mm.

**Figure 11:**
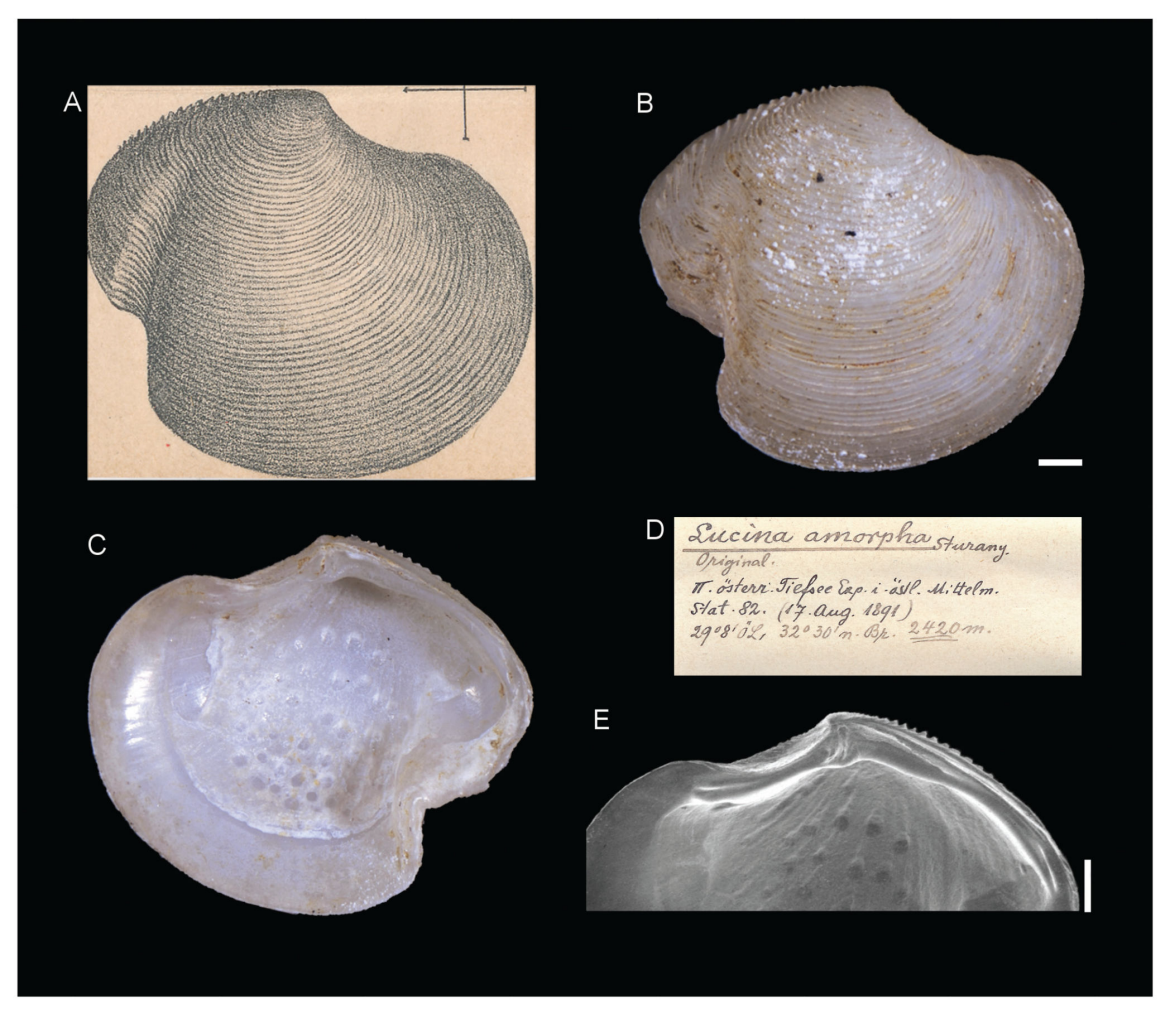
*Lucina amorpha* Sturany, 1896, station 82, north of Alexandria, Egypt, 2420 m. **A**. Original figure in Sturany, 1896. **B**, **C**, **E**. Holotype NHMW 13008: outer (**B**) and inner (**C**) views, hinge (**E**). **D**. Original label. Scale bar: 1 mm.

**Figure 12:**
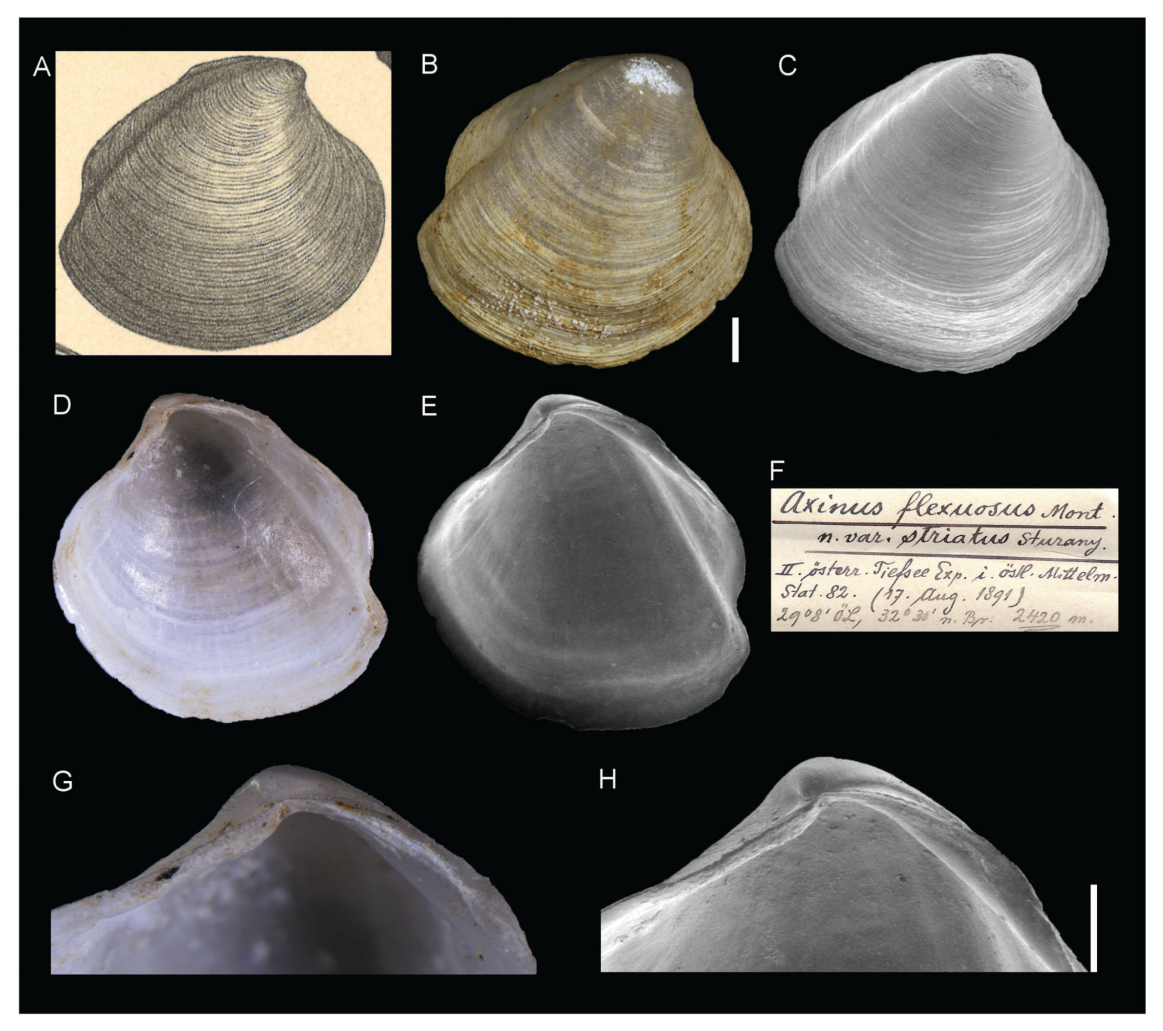
*Axinus flexuosus* var. *striatus* Sturany, 1896, station 82, north of Alexandria, Egypt, 2420 m. **A**. Original figure in Sturany, 1896. **B–E**, **G**, **H**. Holotype NHMW 13009: outer (**B–C**) and inner (**D–E**) views, hinge (**G–H**). **F**. Original label. Scale bar: 1 mm.

**Figure 13:**
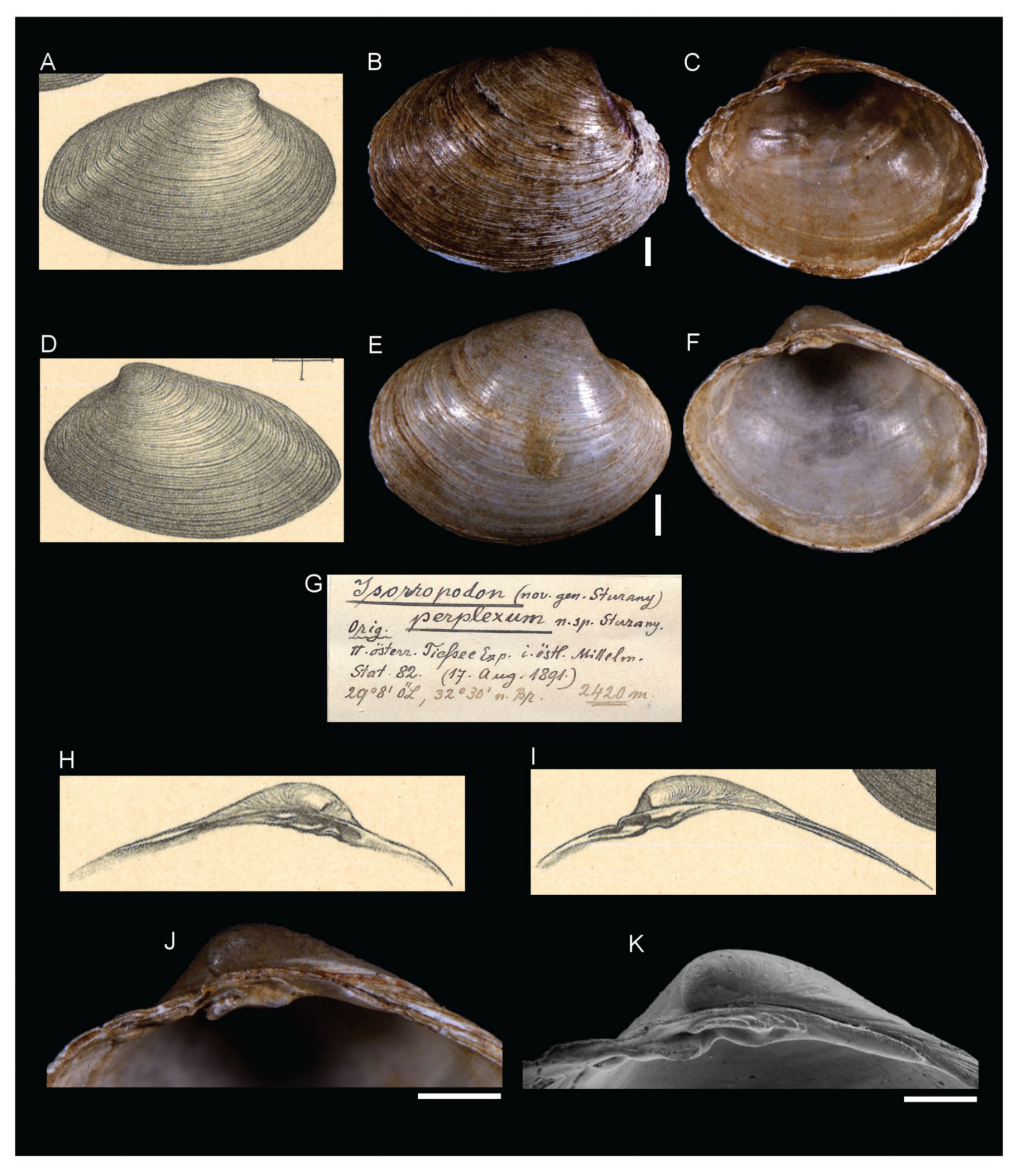
*Isorropodon perplexum* Sturany, 1896, Station 82, north of Alexandria, Egypt, 2420 m. **B–C**, **K**. Syntype NHMW 13010a: outer (**B**) and inner (**C**) views, hinge (K). **E**–**F**, **J**. Syntype NHMW 13010b: outer (**E**) and inner (**F**) views, hinge (**J**). **G**. Original label. **A**, **D**, **H–I**. Original figures in Sturany, 1896. Scale bar: 1 mm.

Sturany 1896: 11-12, plate I, figure 8–9.

**Type locality.** Station 82, north of Alexandria, Egypt, 32°30'N, 29°8'E, 2420 m.

**Type material.** Holotype: NHMW 13002, height 3.40 mm.

**Original description.** *Von Station 82 (2420 m); 1 Schale mit unfertiger Mündung*.

*Gehäuse hellbraun, ziemlich dickschalig, aus 5 Umgängen bestehend. Das Embryonalgewinde (1 ½ Windungen) hat eine etwas rauhe Oberfläche, aber keine deutliche Sculptur. Ziemlich unvermittelt beginnt auf der zweiten Umdrehung die von da ab bis zur Mündung scharf ausgeprägte Querrippung. Die Querrippen sind eng aneinandergelagert, durch einen ober der Mitte verlaufenden Spiralkiel gewinkelt und außerdem von einigen Spiralreifen durchkreuzt. Ein solcher, allerdings nur schwach ausgeprägter Spiralreifen zieht gleich unter der Naht dahin; er ist aber nur auf den letzten zwei Umgängen gut kenntlich. Ferner verlaufen auf dem vorletzten Umgange 2 Spiralreifen unter dem Kiele und auf dem letzten Umgang zwischen Kiel und Nabel deren 6 bis 7. Hier entsteht durch die Querrippung und Spiralstreifung ein Maschenwerk aus schiefgestellten Vierecken oder Quadraten, in denen da und dort eine Körnchensculptur angedeutet ist, was sich aber nur bei stärkerer Lupenvergrösserung sehen lässt*.

*Von den erwähnten Querrippen kommen auf je einen Umgang 22 bis 26 zu stehen*.

*Die Mündung, leider schadhaft, hat eine birnförmige Gestalt, einen nach links gedrehten, kurzen, ausgussartigen Stiel und einen vermutlich auch im ausgewachsenen Zustande scharfen, äußeren Rand. Der Spindelrand ist breit nach links ausgeschlagen und bedeckt einen ritzförmigen Nabel fast ganz*.

*Höhe des Gehäuses 3,5, Breite 2, Höhe der Mündung 1,7 mm. Dass das vorliegende Exemplar mit* Taranis cirrata Brugn*. (Kobelt Prodr., p. 137) verwandt und in das seinem Ursprunge nach nordatlantische Genus* Taranis *zu stellen ist, davon bin ich nicht vollständig überzeugt; darüber wird sich wohl noch streiten lassen. Es ist kleiner als* Taranis cirrata *und hat um eine Umdrehung weniger; ferner tritt ein Spiralreifen deutlich als Kiel hervor, während die anderen schwächeren zurücktreten. Im Ganzen stimmt wohl die Anzahl der Spirallinien, ob sie nun kielartig oder nur reifenförmig auftreten, bei beiden Formen überein, und auch die Anzahl der Querrippen dürfte dieselbe sein wie bei* Taranis citrrata*, aber wir sehen in den genannten abweichenden Merkmalen und besonders in der Gestaltung der Spindel wichtige Unterschiede*.

**Translation.** From station 82 (2420 m); 1 specimen with incomplete aperture.

Light brown shell, fairly thick, consisting of 5 whorls. The protoconch (1 ½ whorls) has a somewhat rough surface, but no distinct sculpture. Quite suddenly, sharply pronounced axial ribbing appear at the second whorl and continue until the aperture. The axial ribs are embedded closely together and angled by a central spiral keel and, moreover, crossed by several spiral threads. Such threads, though pronounced only weakly, are already visible right beneath the suture; however, they are only well distinguishable on the last two whorls. Furthermore, at the body whorl there are 2 spiral stripes beneath the keel and 6 to 7 on the ultimate whorl between keel and umbilicus. As a result of the axial ribbing and spiral stripes, there is a network of slanted rectangles or squares with a hint of a granular sculpture here and there, which is only visible at higher magnification.

Each whorl is equipped with 22 to 26 of the previously mentioned axial ribs.

The unfortunately damaged aperture is pear shaped and has a leftwards rotated, short, nozzle-shaped siphon and a, likely also in the mature state, sharp outer edge. The columella is broadly lashed out towards the left and covers a scar-formed umbilicus almost completely.

Height of the shell 3.5, width 2, aperture height 1.7 mm. I am not entirely convinced that the present specimen is related to *Taranis cirrata* Brugn. (Kobelt Prodr., p. 137) and according to its origin must be placed within the north Atlantic genus *Taranis*. Perhaps this will be further discussed. It is smaller than *Taranis cirrata* and has one whorl less. Furthermore, a spiral stripe is distinctly pronounced as a keel, while the other fainter ones recede. On the whole, probably only the number of spiral lines concur in both forms, whether they appear as a keel or only ring-formed, and also the number of axial ribs seems to be the same as in *Taranis cirrata*, however, we see important differences in the aforementioned traits and especially in the shaping of the columella.

**Comments.** Considered a junior synonym of *Taranis moerchii* (Malm, 1861) (Bouchet and Warén 1980).

**Family Mytilidae Rafinesque, 1815** 

***Myrina modiolaeformis* Sturany, 1896**

Fig. 10

Sturany, 1896: 20, plate II, figures 34–38.

**Original locality.** Station 82, north of Alexandria, Egypt, 32°30'N, 29°8'E, 2420 m.

**Type material.** NHMW 13011: 2 syntypes (1 right and 1 left valve), station 82.

**Original description.** *Von Station 82 (2420 m)*.

*Es liegt nur eine rechte Schale von 13 mm Länge, 6,7 mm Höhe und 3,2 mm Breite (Dicke) vor, sowie von einem zweiten bedeutend kleineren (ungefähr nur halb so großen) Exemplare die linke Schalenhälfte (Länge 6,8, Höhe 3,5 mm). Die Schalen sind aussen bis auf den Wirbel, der sich weisslich abhebt, nahezu einfarbig hellgelb, innen etwas perlmutterglänzend. Die concentrische Streifung der Aussenseite ist etwas unregelmässig, indem einzelne Streifen stärker hervortreten. Der Wirbel liegt weit nach vorne gerückt, im ersten Fünftel oder Sechstel der Schale. Der hintere Oberrand verläuft bis zu seinem gerundeten Übergang in den Hinterrand ganz gerade. Rückwärts bedeutend höher als vorne gebaut, hat die Schale in ihren Umrissen ungefähr die Gestalt von* Modiola (Gregariella) sulcata Risso.

*Was mich veranlasste, diese höchst eigenthümlich gestaltete neue Muschel zu dem exotischen Genus* Myrina *zu beziehen, ist die Querstrichelung auf der inneren Schlossleiste, wodurch diese wie mit einer grossen Anzahl kleiner, senkrecht gestellter und dicht aneinander gereihter Zähnchen besetzt erscheint. Die grosse rechte Schalenhälfte weist nur hier und dort die verwischten Spuren dieser zahnartigen Striche auf, während sie die linke Schale des zweiten Exemplares unter stärkerer Lupenvergrösserung deutlich erkennen lässt, Hier sind die Querstriche nicht blos auf die hintere Schlossleiste beschränkt, sondern stehen auch direkt unter dem Wirbel auf einem zahnartigen Vorsprung, also ungefähr dort, wo der vordere Oberrand entspringt, ganz ähnlich wie bei der im Challenger-Werk (E. Smith, Lamellibr., pl. XVI, fig. 9) abgebildeten* Myrina coppingeri *Wats. von Nord-Australien*.

*Die Zugehörigkeit der hier beschriebenen Muschel zu dem Genus* Myrina *ist trotz der angeführten Schlossmerkmale nichts weniger a1s erwiesen und hierrüber sicher noch nicht das letzte Wort gesprochen*.

**Translation.** From station 82 (2420 m).

There is only one right valve of 13 mm in length, 6.7 mm in height and 3.2 mm in width (thickness), as well as a left valve of a remarkably smaller (approximately only half the size) specimen (length 6.8, height 3.5 mm). The shells are nearly monochrome yellow, except for the umbo which stands out whitish, and shine lightly of mother-of-pearl on the inside. The concentric stripes on the outside are slightly irregular, as single stripes stand out more prominent. The umbo is moved far to the front, within the first fifth or sixth of the shell. The rear upper margin is shaped completely straight until its rounded transition into the posterior margin. Being built significantly higher at the posterior than at the anterior, the shell in its outline has roughly the shape of *Modiola (Gregariella) sulcata* Risso. The axial sketchy lining at the hinge margin, which makes it appear as a set with a large number of smaller, vertically and tightly arranged teeth, caused me to place this very peculiarly shaped new bivalve within the exotic genus *Myrina*. The large right valve shows smeared tracks of these teeth-like lines only here and there, while they are clearly visible under higher magnification in the left valve of the second specimen. Here, the axial lines are not limited to the posterior part of the hinge plate, but are also visible directly beneath the umbo on a tooth-like structure, approximately at the position of the beginning of the anterior upper margin, very similar to *Myrina coppingeri* Wats from northern Australia, depicted in the Challenger work (E. Smith, Lamellibr., pl. XVI, fig. 9).

In spite of the mentioned hinge characters, the belonging of the described bivalve to the genus *Myrina* is no less than evident and the last word has certainly not been spoken yet.

**Comments.** A valid species placed in genus *Idas* (MolluscaBase 2017) recently recorded from cold seep communities in the deep eastern Mediterranean Sea (Olu-Le Roy et al. 2004).

**Family Lucinidae J. Fleming, 1828**

***Lucina amorpha* Sturany, 1896**

Fig. 11

Sturany, 1896: 16, plate I, figure 22.

**Type locality.** Station 82, north of Alexandria, Egypt, 32°30'N, 29°8'E, 2420 m.

**Type material.** Holotype: NHMW 13008 (a right valve), height 9.58 mm, length 10.40 mm.

**Original description.** *Von Station 82 (2420 m); eine rechte Schalenhälfte*.

*Die vorliegende Schale scheint auf den ersten Blick wohl der* Lucina spinifera *Mont. (Kobelt Prodr. p. 369; Carus Prodr. p. 152) anzugehören und ein deformiertes Exemplar zu sein unterscheidet sich aber doch wesentlich in folgenden Punkten:*

*1. Das Möndchen (lunula) ist hier eine schmale, aber tiefe Grube; daher der Umriss der Schale ein ganz anderer als bei* Lucina spinifera*. 2. An der Grenze von Unterund Hinterrand schneidet eine breite winkelige Bucht tief in die Schale und setzt sich bis zur Mitte der Schalenhöhe radial als Konkavität fort (ähnlich wie im Genus* Axinus*). Im Inneren der Schale entspricht dieser Einsenkung von außen eine bauchige Verdickung. 3. Die Anzahl der konzentrischen Streifen (Rippen), welche schwächer sind und näher aneinander gerückt stehen als bei* L. spinifera*, beträgt ca. 66 (gegen ca. 40 bei* L. spinifera*). 4. Der hintere Oberrand zieht vom Wirbel in leichtem konvexen Bogen nach hinten und unten und lässt eine Reihe von schwachen Höckern, die Endigungen der konzentrischen Streifen, erkennen. Doch endigt nicht jeder Streifen, sondern etwa bloß jeder zweite mit einem solchen Höcker*.

*Das Schloss stimmt vollständig mit dem von* L. spinifera *überein, und auch der Wirbel ist wie dort glatt und nach vorne und innen geneigt. Die Farbe ist nahezu rein weiß, die Wölbung der Schale eine schwache. Die Länge der Schale beträgt 11, die Höhe 9,5 mm*.

**Translation.** From station 82 (2420 m); one right valve.

At a first glance, the shell at hand seems to be perhaps a deformed specimen belonging to *Lucina spinifera* Mont. (Kobelt Prodr.p. 369; Carus Prodr. p. 152), but is after all clearly distinguished regarding the following points:

1. The lunula, in this case, is a narrow but deep cavity, therefore the outline of the shell is quite a different one than in *Lucina spinifera*. 2. At the boundary between ventral and posterior margin, a broad, cornered cove cuts deep into the shell and continues radially as a concavity (similar to the genus *Axinus*) further to the middle of the shell height. On the inside of the shell, this depression corresponds to a bulbous thickening from the outside. 3. The number of concentric ridges, which are fainter and stand closer together than in *L. spinifera*, amounts to approximately 66 (compared to about 40 in *L. spinifera*). 4. The posterior upper margin runs from the umbo back- and downwards in a slightly convex curve and bears a row of faint humps at the endings of the concentric lines. However, not each one of these lines, but only approximately every second one, ends in such a hump.

The hinge is identical to that of *L. spinifera*, and also the umbo is smooth and tilted forwards and inwards as it is there. The colour is nearly clear white, the curvature of the shell is weak. The length of the shell is 11, the height 9.5 mm.

**Comments.** Currently accepted as *Myrtea amorpha* (Sturany, 1896) (MolluscaBase 2017) and recently recorded from cold seep communities in the deep eastern Mediterranean Sea (Olu-Le Roy et al. 2004). Its shape is totally unusual for lucinids and is indeed due to an accident during growth.

**Family Thyasiridae Dall, 1900**

***Axinus flexuosus* var. *striatus* Sturany, 1896**

Fig. 12

Sturany 1896: 17, plate I, figures 23.

**Type locality.** Station 82, north of Alexandria, Egypt, 32°30'N, 29°8'E, 2420 m.

**Type material.** Holotype: NHMW 13009 (a right valve), height 7.09 mm, length 7.37 mm.

**Original description.** *Von Station 82 (2420 m.); eine rechte Schalenhälfte*.

*Die stärkere concentrische Streifung der Schale, die noch weiter als im Typus nach vorne gerückte Stellung des Wirbels und der weniger gewölbte Unterrand zwingen zur Abtrennung der Form als eine Varietät von* Axinus flexuosus *Mont. und verrathen auch eine gewisse Verwandtschaft mit der bisher nur im Norden gefundenen* Axinus sarsii *Phil. – Die Länge der Schale beträgt 7,5, die Höhe 7 mm*.

**Translation.** From station 82 (2420 m); one right valve.

The shells stronger concentric stripes, the position of the umbo which is moved further to the front than in the type specimen, and the lesser arched ventral margin require a separation of this form as a variety of *Axinus flexuosus* Mont. and also suggest a certain relation to *Axinus sarsii* Phil., which was only found in the north to this date – the length of the shell is 7.5, the height 7 mm.

**Comments.** Currently accepted as *Thyasira striata* Sturany, 1896 (MolluscaBase 2017) and recently recorded from cold seep communities in the deep eastern Mediterranean Sea (Olu-Le Roy et al. 2004).

**Family Vesicomyidae Dall & Simpson, 1901**

***Isorropodon perplexum* Sturany, 1896**

Fig. 13

Sturany 1896: 17, plate I, figures 24–27.

**Original locality.** Station 82, north of Alexandria, Egypt, 32°30'N, 29°8'E, 2420 m.

**Type material.** NHMW 13010: 11 syntypes (3 left and 8 right valves), station 82.

**Original description.** *Von Station 82 (2120 m)*

*Es liegt von dieser rätselhaften*, *neuen Muschel eine größere Anzahl linker und rechter Schalenhälften*

*vor, die in ihren Massverhältnissen ziemlich verschieden, also durchaus nicht konstant sind und von denen kaum zwei zusammenpassen. Von Farbe sind die Schalen außen weiß, gelb oder braun, innen weißlich mit gelbem Saume am Rande oder einfarbig gelblich bis grau ohne Saum. Vorne und rückwärts sind sie zumeist gleichmässig abgerundet, seltener lässt sich rückwärts die schwache Andeutung eines schnabelförmigen Endes konstatieren, indem der Unterrand an seinem Übergange in den Hinterrand eine leichte Einbuchtung zeigt. Die Außenseite der Schale ist dicht concentrisch gestreift; die Innenseite, glatt und glänzend, weist zwei längliche senkrecht stehende Muskeleindrücke auf und rückwärts eine sehr seichte Mantelbucht. Der Wirbel steht in der vorderen Hälfte der Schale und neigt sich mit seiner Spitze nach vorne und innen; vor demselben ist eine lunula-artige Vertiefung wahrzunehmen*.

*Das Schloss ist ziemlich kompliziert. In der rechten Schale (Fig. 26) steht direct unter dem Wirbel ein waagrechter, von oben nach unten komprimierter, langgestreckter Zahn, und vor dem Wirbel, nämlich unter dem vorderen Oberrande und von diesem durch eine Rinne getrennt, ein zweiter, ebenfalls waagrechter und abgeflachter Zahn. Die beiden Zähne sind wohl an ihrer Basis miteinander verbunden, lassen aber oben, resp. an der nach dem Innern der Schale gekehrten Partie eine Höhlung zwischen sich. Hinter dem Wirbel verläuft eine Leiste parallel mit dem Oberrande*.

*In der linken Schale (Fig. 27) fällt dem Auge des Beschauers sofort eine grosse Zahnpartie auf, welche unter dem Wirbel steht und, senkrecht zur Längsebene der Schale betrachtet, zwei mit ihren konvexen Seiten aneinander gelehnte Bogen erkennen 1ässt, wodurch sie das Aussehen eines Doppelzahnes gewinnt. Diese Zahnpartie kommt beim Schließen der Muschel jedenfalls in die Höhlung zu liegen welche sich zwischen den beiden großen Zähnen der rechten Schale ausdehnt, und zwar dürfte sich an jeden derselben einer der 2 oben erwähnten Bögen anlegen. Zwischen dem „Doppelzahne“ und dem unter dem Wirbel flächenartig verbreiterten Oberrande liegt analog dem Verhalten in der rechten Schale eine Rinne und über derselben, also direkt am Oberrande ein kleiner abgeplatteter Zahn oder – was aber nur bei einem Exemplar der Fall ist – 2 waagrechte und einander parallel gestellte Zähnchen. Auch in der linken Schale zieht hinter dem Wirbel eine stellenweise fast zahnartig vorspringende Leiste parallel zum hinteren Oberrande dahin*.

*Das derartig beschaffene Schloss ist ähnlich dem von* Cypricardia lithophagella *Lam. (Kobelt Prodr. p. 390), und mit Rücksicht darauf halte ich es für passend, das neue Genus* Isorropodon*. welches mit der obigen, vorläufig allerdings nur nach der einzigen vorliegenden Art entworfenen Beschreibung charakterisiert ist, im Systeme unmittelbar hinter* Cypricardia *zu stellen*.

[A table with the sizes of the collected specimens follows]

**Translation.** From station 82 (2420 m)

There is a large number of right and left valves of this mysterious new bivalve, which are quite diverse in their sizes and of which scarcely any fit together.

The color on the outside of the shell is white, yellow, or brown and whitish on the inside with a yellow margin or monochrome yellowish to gray with no margin. Anterior and posterior sides are in most cases equally rounded. In some rarer cases, there is a faint suggestion of a beakformed ending, showing a slight dent at the transition of the ventral margin to the posterior margin. The outer surface of the shell is densely concentrically lined; the inside, smooth and shiny, shows two elongated vertical muscle scars and a very superficial/shallow pallial sinus at the posterior. The umbo is located at the anterior half of the shell and its tip tilted to the front and inside. At its front, a lunular-like indentation can be noticed.

The hinge is quite complex. In the right valve (Fig. 26), directly beneath the umbo there is an horizontal elongated tooth, compressed from top to bottom. In front of the umbo, namely beneath the anterior lower margin and separated by it through a groove, there is a second, also horizontal and flattened tooth. The two teeth are connected at their base, but leave a cavity above, respectively at the inwards turned part, between them. Behind the umbo, a bar advances parallel to the upper margin.

In the left valve (Fig. 27), a large set of teeth beneath the umbo strikes the viewer. Perpendicularly to the longitudinal plane of the shell, two curves become visible, leaning together at their convex sides, which makes the teeth seem double-toothed. When the shell closes, this set of teeth is embedded in a caving which is stretched between the two large teeth of the right valve and it seems as the aforementioned curves dock each of them. Between the “double tooth” and the upper margin which is broadened beneath the umbo, there is a groove and above, directly at the upper margin, a small flattened tooth, analogous to the right valve. Only in one specimen we find two horizontal and parallel arranged teeth instead. Also in the left valve there is a bar advancing behind the umbo parallel to the posterior upper margin which is prominent and tooth-like in some parts.

The hinge of this sort is similar to that of *Cypricardia lithophagella* Lam. (Kobelt Prodr. p. 390), and considering this, I find appropriate to introduce the new genus *Isorropodon* which is characterized by the described hinge and stands in the system directly behind *Cypricardia*.

**Comments.** The genus *Isorropodon* Sturany, 1896 was also introduced and is currently considered a valid genus within the vesicomyid subfamily Pliocardiinae (Krylova and Sahling 2010). *I. perplexum* is considered a valid specis (MolluscaBase 2017) and has been recently recorded from cold seep communities in the deep eastern Mediterranean Sea (Olu-Le Roy et al. 2004). Types noted and species described in detail by von Cosel and Salas (2001).

**Family Lyonsiidae P. Fischer, 1887** 

***Lyonsia aegeensis* Sturany, 1896**

Fig. 14

**Figure 14:**
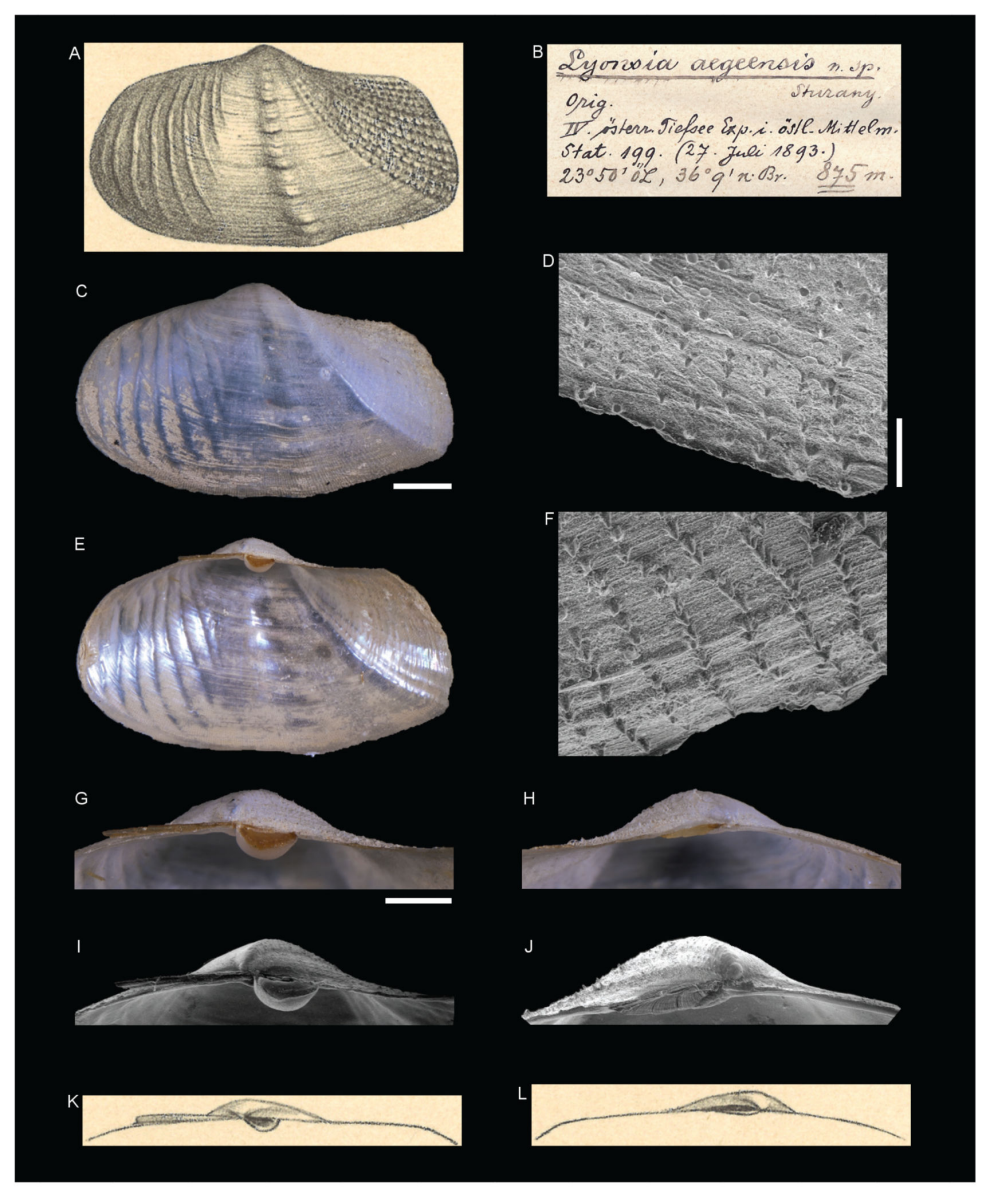
*Lyonsia aegeensis* Sturany, 1896, Station 199, southwest of Kythira, Sea of Crete, Greece, 875 m. **A**, **K**, **L**. Original figures in Sturany, 1896. **B**. Original label. **C–J**. Syntype NHMW 13004: outer view of left valve (**C**), inner view of right valve (**E**), microsculpture (**D**, **F**), hinge right valve (**G**, **I**) and left valve (**H**, **J**). Scale bar: 2 mm (**C, E**), 0.2 mm (**D, F**), 1 mm (**G–J**).

**Figure 15:**
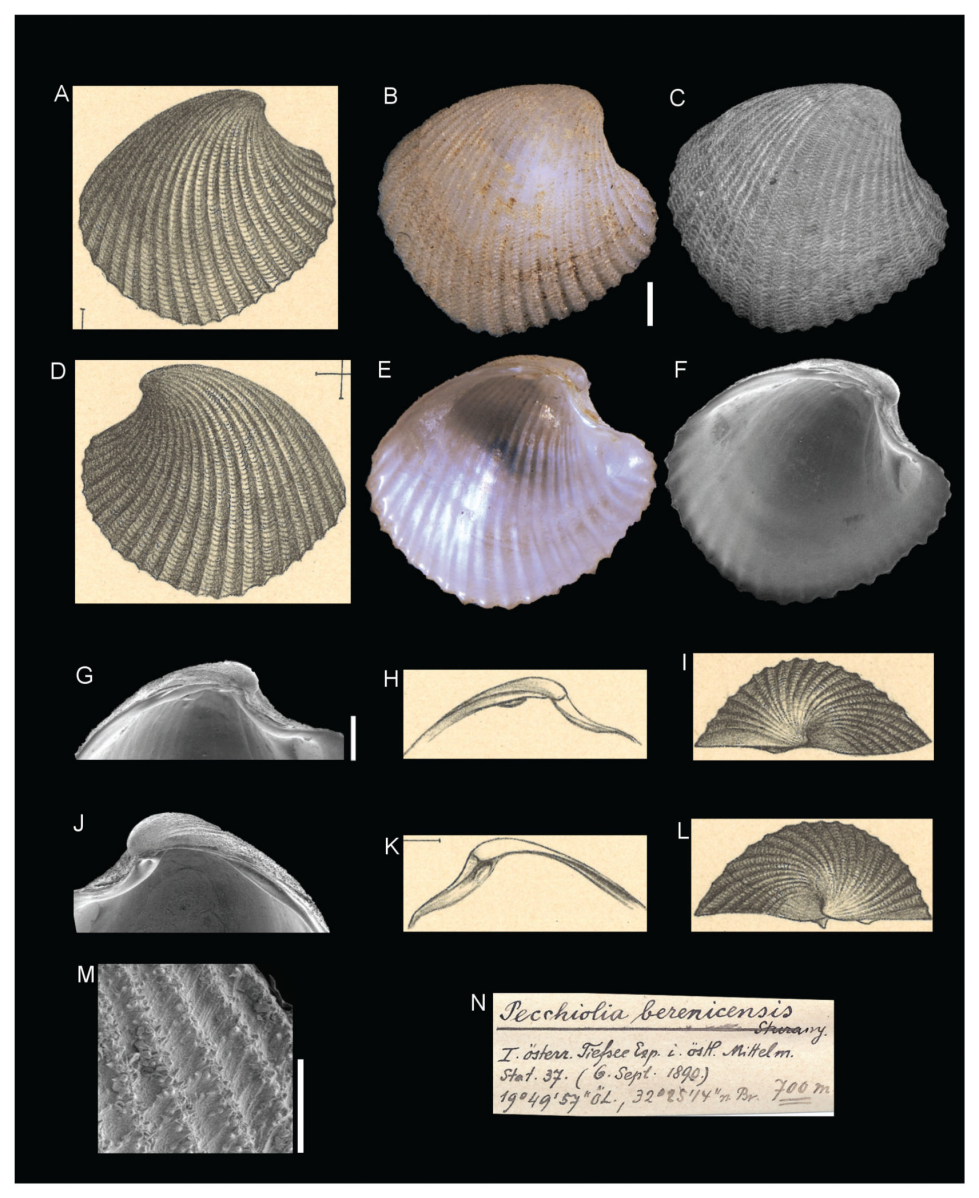
*Pecchiolia berenicensis* Sturany, 1896, Station 37, north of Benghazi, Libya, 700 m. **A**, **D**, **H–I**, **K–L**. Original figures in Sturany, 1896. **B–C**, **E–G**, **J**, **M**. Holotype NHMW 13007: outer view left valve (**B–C**), inner view right valve (**E–F**), hinge detail of right (**G**) and left valve (**J**), sculpture detail (**M**). **N**. Original label. Scale bar: 1 mm (**B–C, E–F, G, J**), 0.5 mm (**M**).

Sturany 1896: 15, plate I, figures 14–16.

**Original localities**. Station 199, southwest of Kythira, Sea of Crete, Greece, 36°9'N, 23°50'E, 875 m; Station 213, north of Astypalaia, Dodekanese, Greece, 36°47'N, 26°29'E, 597 m; Station 237, southwest of Samothraki, Aegean Sea, Greece, 40°17'N, 25°13'E, 588 m.

**Type material.** NHMW 13004: 1 syntype (a complete specimen), station 199; NHMW 13005: 1 syntype (a right valve), station 237; NHMW 13006: 1 syntype (3 fragments), station 213.

**Original description.** Von den Stationen *199 (875 m, 1 vollständiges Exemplar), 213 (597 m, Fragment) und 237 (588 m, 1 rechte Schalenhälfte).

*Das vollständige Exemplar von Station 199 ist 17 mm lang, 10 mm hoch und 7,2 mm breit. Es ist sehr nahe mit* Lyonsia formosa *Jeffr. verwandt (Kobelt Prodr. p. 321; Carus Prodr. p. 170; E. Smith, Challenger-Report, Lamellibr., p. 72, pl. VI, fig. 3–3b) und würde sich vielleicht, wenn es möglich wäre, eine Reihe von* Lyonsien *vergleichend zu studieren, bloss als eine Varietät von* L. formosa *erweisen. Vorläufig aber muss ich die vorliegende Form isolieren, da sie im Vergleiche zu dem im Challenger-Werke abgebildeten* formosa*-Exemplare in folgenden Punkten abweicht:*

1. *Die Größe der Schale ist eine viel bedeutendere, die allgemeine Form eine gestrecktere. 2. Der Wirbel liegt nahezu in der Mitte, während denselben das Challenger-Exemplar (vide d. Ansicht von oben!) mehr gegen das Vorderende gerückt hat. 3. Die vom Wirbel bis ungefähr zur Mitte des Unterrandes ziehende Radialerhöhung ist schwach angedeutet und nur durch eine Reihe blasenförmiger Auftreibungen der Epidermis kenntlich. Der zweite Kiel, welcher vom Wirbel zum hinteren Unterrand der Schale zieht, ist deutlich markiert und beschreibt einen Bogen, dessen Convexität nach vorne zu liegt, während sich beim Challenger-Exemplar dieser Kiel in seinem oberen Theil mit einer schwachen Convexität dem hinteren Oberrande nähert. 4. Hinter dem zweiten Kiele folgen 11 ebenfalls radial ausstrahlende Rippen mit dicht aufsitzenden Dörnchen (gegen 7 beim Challenger-Exemplar!) und in der vorderen Hälfte der Schale kommen zu den für* L. formosa *charakteristischen 6 oder 7 stärkeren Querwülsten noch etliche schwächere, enger aneinander stehende wellenförmige Erhebungen, welche hier denjenigen Raum bis zum Wirbel einnehmen, der am Challenger-Exemplar hiervon frei zu sein scheint. 5. Schließlich wäre noch zu erwähnen, dass der Oberrand mit dem Hinterrande einen deutlichen Winkel bildet, indem er horizontal vom Wirbel ausläuft und mehr plötzlich nach abwärts umbiegt, also nicht, wie dies am Challenger-Exemplar geschieht, langsam und allmählich in den Hinterrand abfällt*.

*Sehr hübsch ist an dem hier besprochenen Exemplar auch das Ligament mit dem halbkugeligen ossiculum zu sehen. Jede Schale trägt unter dem Wirbel eine horizontal etwas hervortretende, oben ausgehöhlte Platte zur Aufnahme des Ligaments samt dem ossiculum*.

*Die Schale von Station 237 misst bis bloß 11 1/2 mm in der Länge und 8 1/2 mm in der Höhe*.

**Translation.** From stations 199 (875 m, 1 complete specimen), 213 (597 m, fragment), and 237 (588 m, 1 right valve).

The complete specimen from station 199 is 17 mm long, 10 mm high and 7.2 mm wide. It is very closely related to *Lyonsia formosa* Jeffr. (Kobelt Prodr. p. 321; Carus Prodr. p. 170; E. Smith,

Challenger-Report, Lamellibr., p. 72, pl. VI, fig. 3–3b) and might turn out to be, if it would be possible to comparably study a range of *Lyonsia*, merely a variety of *L. formosa*. For now, however, I have to isolate the present form, as it differs from the *formosa* specimen depicted in the Challenger work in the following points:

1. The size of the shell is much larger; the general form is more elongated. 2. The umbo is situated nearly in the middle, while it is moved further to the anterior in the Challenger specimen (see view from above). 3. The radial ridge which proceeds from the umbo towards about the middle of the lower margin is faintly indicated and only recognizable through a row of blister-formed swellings of the epidermis. The second ridge, which proceeds from the umbo towards the posterior lower margin of the shell, is clearly marked and describes a curve which is convex to the front, while this ridge is orientated towards the posterior upper margin with a weak convexity in its upper part. 4. There are 11 radially expanding ribs with densely packed spines behind the second keel (as compared to 7 in the Challenger specimen!). In addition to the 6 or 7 more prominent varices which are typical for *L. formosa*, there are several wavy elevations in the anterior half of the shell, which are less developed and positioned closer together. Here, these elevations occupy the space towards the umbo which seems to be open in the case of the Challenger specimen. 5. Finally, it can be mentioned that the upper margin forms a distinct angle with the posterior margin, by ending horizontally from the umbo and suddenly bending downwards, not slowly and gradually bending towards the posterior, as it happens in the Challenger specimen.

The ligament with the hemispheric ossiculum is beautifully visible in the here described specimen. Each shell has a horizontally emerging and upright carved plate beneath the umbo to accommodate the ligament including the ossiculum.

The shell from station 237 is merely 11 ½ mm long and 8 ½ mm high.

**Comments.** Currently considered a junior synonym of *Allogramma formosa* (Jeffreys, 1882) (MolluscaBase 2017).

**Family Verticordiidae Stoliczka, 1870**

***Pecchiolia berenicensis* Sturany, 1896**

Fig. 15

Sturany 1896: 15, plate I, figure 17–21.

**Type locality.** Station 37, north of Benghazi, Libya, 32°25'14"N, 19°49'57"E, 700 m.

**Type material.** NHMW 13007: holotype (a complete specimen), length 6.70 mm, height 6.31 mm.

**Original description.** Von Station *37 (700 m.); 1 Exemplar.

*Diese Muschel ist von weisser Farbe, ungleichschalig, ungleichseitig und in ihren Umrissen von der Gestalt eines schief gestellten, ungleichseitigen Viereckes. Die Wirbel liegen in der vorderen Hälfte der Schale. Der schief abwärts geneigte vordere Oberrand ist muldenförmig vertieft (Abgrenzung der lunula) und geht unter einem rechten Winkel direct in den Unterrand über, welcher stumpfwinkelig ist (mit der Spitze in der Mitte) und rückwärts wieder beiläufig unter einem rechten Winkel in den hinteren, convex gekrümmten Oberrand sich fortsetzt. Von einem Vorder- und Hinterrand ist also hier nicht zu reden*.

*Die rechte Schale übertrifft die linke an Länge und Höhe. Während jene nämlich 7,5 mm in der Länge und 7,1 mm in der Höhe misst, ist diese nur 7,1 mm lang und 6,4 mm breit. Der Rand der überdies auch stärker gewölbten rechten Schale greift, wenn die Muschel geschlossen ist, etwas über den der linken. Die Dicke der ganzen Muschel beträgt 5,5 mm*.

*Über die Aussenseite der beiden Schalenhälften laufen 23 Radialrippen, welche auch an der perlmutterartig glänzenden Innenseite durchscheinen, und zwischen diesen stehen feine, gewellte, concentrisch angeordnete Querlinien in grosser Anzahl*.

*Das Schloss ist ziemlich einfach. In der rechten Schale steht ein starker, konischer Hauptzahn unter dem nach vorne und innen gerichteten Wirbel und rückwärts (unter dem hinteren Oberrande) verläuft eine ziemlich lange, in ihrer mittleren Parthie mässig vorspringende Leiste, welche einen Seitenzahn vorstellt; zwischen beide aber, den Haupt- und Seitenzahn, kommt in eine Vertiefung das hornartige innere Ligament zu liegen. In der linken Schale befindet sich unter dem Wirbel eine Vertiefung zur Aufnahme des Hauptzahnes der rechten Schale, und der hintere Seitenzahn ist hier wieder in Form einer Leiste vorhanden, die aber soweit gegen den Wirbel zu gerückt ist, dass das hornige innere Ligament gerade darunter zu liegen kommt. Ist die Muschel geschlossen, so steht der Seitenzahn der linken Schale gerade vor dem der rechten, und unter dem ersteren liegt, wie gesagt, das Ligament*.

*An der Bildung des Möndchens (lunula), welches breit herzförmig ist und sehr vertieft liegt, betheiligen sich die beiden Schalen nicht in gleichem Masse. Der vordere Oberrand der rechten Schale ist an der betreffenden Stelle convex vorgezogen und passt in eine entsprechende Concavität des gegenüberliegenden Randes; die von der rechten Schale gelieferte Fläche des Möndchens ist also grösser als die linke*.

*Die vorliegende Art fällt wohl mit keiner der aus dem Genus* Verticordia *oder* Pecchiolia *bisher bekannt gewordenen zusammen, ist aber mit* Pecchiolia insculpta *Jeffr. (P. Z. S. 1881, p. 932, pl. 70, fig. 4.- Kobelt Podr. p. 323; Carus Prodr. p. 165.) aus dem Atlantischen Ocean und dem westlichen Mittelmeere nahe verwandt*.

**Translation.** From station 37 (700 m); 1 specimen.

This bivalve is white in colour, with a rough surface, unequilateral, and rhomboid in outline. The umbos are located at the anterior half of the shell. The downwards tilted dorsal margin is depressed (delimiting the lunula) and merges through a right angle directly with the lower margin which has an obtuse angle (with the tip in the middle). Posteriorly, the margin runs again through a right angle towards the posterior, convex bended upper margin. Therefore, there cannot even be any question of an anterior or posterior margin.

The right valve surpasses the left one in length and height. In fact, while that [right] valve measures 7.5 mm in length and 7.1 mm in height, this [left] one is only 7.1 mm long and 6.4 mm wide. The margin of the right valve, furthermore also more strongly arched, slightly surpasses the margin of the left valve when the shells are closed. The thickness of the entire bivalve is 5.5 mm.

There are 23 radial ribs running along the outside of the two valves, which show through the mother-of-pearl shining inside. Between them, there are a large number of delicate, waved concentrically arranged lateral lines.

The hinge is rather ordinary. In the right valve there is a strong, conic cardinal tooth beneath the front- and inwards orientated umbo; proceeding backwards (beneath the posterior upper margin) there is a rather long, in the mid part moderately projected, bar, which lies before a lateral tooth; embedded between the two, the cardinal and lateral tooth, is the horn-like ligament. Situated within the left valve is a deepening beneath the umbo to accommodate the cardinal tooth of the right valve. The posterior lateral tooth is present here again in the form of a bar, which is moved so far against the umbo that the horny inner ligament is placed right beneath it. Whenever the mussel is closed, the lateral tooth of the left valve is placed right in front of that of the right one and beneath the first one lies the ligament, as mentioned before.

The lunula, which is broad and heart-shaped and is positioned very deep, has not the same size in the two valves. The anterior upper margin of the right valve is convex at the relevant position and fits into an equivalent concavity of the opposite margin; the space of the lunula of the right valve is also larger than the part of the left valve.

The species at hand seem not to coincide with any of recognized species of the genera *Verticordia* or *Pecchiolia* to date, however it is closely related to *Pecchiolia insculpta* Jeffr. (P. Z. S. 1881, p. 932, pl. 70, fig. 4.– Kobelt Podr. p. 323; Carus Prodr. p. 165.) from the Atlantic ocean and the western Mediterranean.

**Comments.** Currently accepted as *Haliris berenicensis* (Sturany, 1896) (MolluscaBase 2017), but its affinity with *Haliris granulata* (Seguenza, 1860) deserves further investigation.

## Figures and Tables

**Table 1:** List of treated taxa in alphabetic order, with original name, current family placement, and figure in this paper.

Taxon	Current name	Family	Page, Figure
*aegeensis, Lyonsia*	*Allogramma formosa* (Jeffreys, 1882)	Lyonsiidae	Page 51, figure 14
*alexandrina, Taranis*	*Taranis moerchii* (Malm, 1861)	Raphitomidae	Page 44, figure 9
*amorpha, Lucina*	*Myrtea amorpha* (Sturany, 1896)	Lucinidae	Page 47, figure 11
*bengasiensis, Fusus*	*Fusinus rostratus* (Olivi, 1792)	Fasciolariidae	Page 38, figure 5
*berenicensis, Pecchiolia*	*Haliris berenicensis* (Sturany, 1896)	Verticordiidae	Page 53, figure 15
*cerigottana, Scalaria*	*Punctiscala cerigottana* (Sturany, 1896)	Epitoniidae	Page 33, figure 3
**corallinoides*, *Raphitoma *nuperrima*** var.	*Kurziella serga* (Dall, 1881)	Raphitomidae	Page 41, figure 8
*igneus, Jujubinus*	*Jujubinus exasperatus* (Pennant, 1777)	Trochidae	Page 30, figure 2
*implicisculpta, Defrancia*	*Pleurotomella eurybrocha* (Dautzenberg & Fischer, 1896)	Raphitomidae	Page 40, figure 7
*minor, Marginella occulta* var.	–	Marginellidae	Page 38, figure 6
*modiolaeformis, Myrina*	*Idas modiolaeformis* (Sturany, 1896)	Mytilidae	Page 45, figure 10
*perplexum, Isorropodon*	*Isorropodon perplexum* (Sturany, 1896)	Vesicomyidae	Page 48, figure 13
*pianosana, Fusus craticulatus var*.	*Hirtomurex squamosus* (Bivona Ant. in Bivona And., 1838)	Muricidae	Page 36, figure 4
*pseudacanthodes, *Raphitoma *nuperrima*** var.	*Kurziella serga* (Dall, 1881)	Raphitomidae	Page 41, figure 8
*ruderatus, Pseudomurex*	*Hirtomurex squamosus* (Bivona Ant. in Bivona And., 1838)	Muricidae	Page 38, figure 4
*striatus, Axinus flexuosus* var.	*Thyasira striata* Sturany, 1896	Thyasiridae	Page 47, figure 12

**Table 2:** Stations of the “Pola” expeditions to the Eastern Mediterranean Sea listed by Sturany (1896) where molluscs were collected.

Station number	Locality	Coordinates	Depth [m]
1	“Westlich von Corfu” [west of Corfu, Greece]	39°23'N, 19°48'20"E	-615
9	“Vor der Bucht von Kalamata (Griechenland)” [off the Bay of Kalamata, Greece]	36°38'55"N, 22°4'36"E	-1050
19	“Südlich von Cerigo” [south of Kythira, Ionian Sea, Greece]	35°56’N, 22°54'50"E	-1010
27	“An der afrikanischen Küste” [off the African coast, NE of Benghazi]	33°11'18"N, 22°22'56"E	-1765
36	“Nördlich von Benghazi an der afrikanischen Küste” [north of Benghazi, Libya]	32°46'40"N, 19°58'30"E	-680
37	“Nordwestlich von Benghazi an der afrikanischen Küste” [north of Benghazi, Libya]	32°25'14"N, 19°49'57"E	-700
62	“Im Norden der Westküste von Creta” [north of the west coast of Crete, Greece]	35°48'N, 23°34'E	-755
64	“Südwestlich von der Insel Cerigo” [southwest of the island of Kythira, Ionian Sea, Greece]	35°59'N, 22°56'E	-660
70	“Vor der Plaka-B. von Creta (Candia)” [off the Bay of Plaka (north of Vamos) in Crete, Chania, Greece]	35°39'N, 24°23'E	-805
73	“Nördl. Ausgang des Hafens von Santorin (Cycladen)” [northern entrance of the harbour of Santorini (Cyclades), Greece]	36°26'N, 25°24'E	-381
82	“Nördlich von Alexandrien” [north of Alexandria, Egypt]	32°30'N, 29°8'E	-2420
103	“Südlich vom Cap St. Maria di Leuca (Jon. Meer) [south of St. Maria di Leuca Cape, Ionian Sea, Italy]	39°54'N, 18°44'E	-134
194	“Zwischen Cerigo und Cerigotto” [between Kythira and Antikythira, Ionian Sea, Greece]	36°3'N, 23°6'E	-160
197	“Zwischen Cerigo und Candia (Creta)” [between Kythira and Chania (Crete), Greece]	35°45'N, 23°11'E	-608
199	“Südöstlich von Cerigo (Meer von Candia)” [southwest of Kythira, Sea of Crete, Greece]	36°9'N, 23°50'E	-875
200	“Mitten zwischen Cap Malea und Santorin (Meer von Candia)” [between Cape Maleas and Santorini (Sea of Crete), Greece]	36°23'N, 24°11'E	-880
204	“Zwischen Cap Malea und Milo (Meer von Candia)” [between Cape Maleas and Milos, Sea of Crete, Greece]	36°25'N, 24°2'E	-808
208	“Mitten zwischen Milo und Serpho (Cycladen)” [between Milos and Serifos (Cyclades), Greece]	37°0'N, 24°28'E	-414
209	“Ebenda” [as above]	36°59'N, 24°29'E	-444
210	“Östlich von Serpho (Cycladen)” [east of Serifos (Cyclades), Greece]	37°12'N, 24°43'E	-287
213	“Nördlich von Stampaglia (Astropalia), Sporaden” [north of Astypalaia, Dodekanese, Greece]	36°47'N, 26°29'E	-597
214	“Östlich von Stampaglia, Sporaden” [east of Astypalaia, Dodekanese, Greece]	36°37'N, 26°43'E	-533
227	“Ebenda” [as above]	37°37'N, 26°58'E	-92
229	“Nördlich von Samos” [north of Samos, Greece]	37°54'N, 26°43'E	-580
237	“Südwestlich von Samotraki (Äg. M.) [southwest of Samothraki, Aegean Sea, Greece]	40°17'N, 25°13'E	-588
238	“Nördlich von Tremiti” [north of Tremiti Islands, Adriatic Sea, Italy]	42°2'40"N, 15°27'7"E	-98
239	“Ebenda” [as above]	-	-70
240	“Zwischen Tremiti und Pianosa” [between Tremiti Islands and Pianosa Isl., Adriatic Sea, Italy]	42°9'N, 15°22'37"E	-104
243	“In der Linie von Tremiti und Pianosa” [between Tremiti Islands and Pianosa Isl., Adriatic Sea, Italy]	42°11'40"N, 15°40'50"E	-103
247	“Bei Pianosa” [Pianosa Isl., Tremiti Islands, Adriatic Sea, Italy]	42°13'20"N, 15°50'42"E	-111
251	“Vor Pelagosa” [off Pelagruža, Croatia]	42°23'24"N, 16°1'42"E	-129
255	“Bei Pelagosa” [at Pelagruža, Croatia]	42°34'18"N, 16°9'15"E	-176
259	“Bei Pelagosa” [at Pelagruža, Croatia]	42°23'40"N, 16°20'45"E	-174
260	“Bei Pelagosa” [at Pelagruža, Croatia]	42°23'3"N, 16°21'50"E	-128
261	“Bei Pelagosa” [at Pelagruža, Croatia]	42°23'8"N, 16°12'42"E	-101
267	“Bei Lagosta” [at Lastovo, Croatia]	42°9'0"N, 15°22'37"E	-117
271	Not specified	42°2'0"N, 15°27'7"E	-112
274	Not specified	42°31'44"N, 16°27'50"E	-191
279	“Bei Cazza” [at Sušac, Croatia]	42°47'0"N, 16°21'10"E	-132
283	“Zwischen Lissa und Busi” [between Vis and Biševo, Croatia]	42°58'24"N, 16°3'24"E	-102
284	“Zwischen Comisa und Busi” [between Komiža and Biševo, Croatia]	43°2'24"N, 16°0'10"E	-94
285	“Zwischen St. Andrä und Lissa” [between Sveti Andrija and Vis, Croatia]	42°58'20"N, 15°43'10"E	-133
292	Not specified	42°24'44"N, 16°17'42"E	-171
293	“Östlich von Pelagosa” [east of Pelagruža, Croatia]	42°23'0"N, 16°21'59"E	-131
298	“Südöstlich von Pelagosa” [southeast of Pelagruža, Croatia]	42°9'0"N, 16°21'27"E	-485
301	“Südöstlich von Pelagosa” [southeast of Pelagruža, Croatia]	42°11'0"N, 17°51'30"E	-1216
315	“Strasse von Otranto, in der Höhe von Valona” [Strait of Otranto, off Valona, southern Adriatic Sea]	40°40'20"N, 18°51'30"E	-840
316	“Strasse von Otranto” [Strait of Otranto, southern Adriatic Sea]	40°32'45"N, 18°58'0"E	-760
318	“Strasse von Otranto, nach der Ausfahrt von Valona” [Strait of Otranto, at the entrance of Valona, southern Adriatic Sea]	40°13'10"N, 19°3'40"E	-932
365	“Strasse von Otranto” [Strait of Otranto, southern Adriatic Sea]	between 40°46'6"N, 19°3'E and 40°36'N, 18°31'E	-776
378	“Südliche Adria” [southern Adriatic Sea]	41°36'8"N, 17°35'7"E	-950
379	“Südliche Adria” [southern Adriatic Sea]	41°41'N, 17°30'5"E	-1138
385	“Südliche Adria” [southern Adriatic Sea]	41°37'N, 17°38'E	-1196
389	“Südliche Adria” [southern Adriatic Sea]	41°42'N, 18°5'40"E	-1205
399	“Südlich von Meleda” [south of Mljet, Croatia]	42°32'20"N, 17°28'40"E	-218
